# Determination of system level alterations in host transcriptome due to Zika virus (ZIKV) Infection in retinal pigment epithelium

**DOI:** 10.1038/s41598-018-29329-2

**Published:** 2018-07-25

**Authors:** Pawan Kumar Singh, Indu Khatri, Alokkumar Jha, Carla D. Pretto, Katherine R. Spindler, Vaithilingaraja Arumugaswami, Shailendra Giri, Ashok Kumar, Manoj K. Bhasin

**Affiliations:** 1 Department of Ophthalmology, Visual and Anatomical Sciences, Wayne State University, Detroit, MI USA; 2 BIDMC Genomics, Proteomics, Bioinformatics and Systems Biology Centre, Beth Israel Deaconess Medical Center, Boston, MA USA; 3 Division of Interdisciplinary Medicine and Biotechnology, Department of Medicine, Beth Israel Deaconess Medical Center, Harvard Medical School, Boston, MA USA; 4 Department of Microbiology and Immunology, University of Michigan, Ann Arbor, MI USA; 5 Department of Molecular and Medical Pharmacology, University of California, Los Angeles, CA USA; 6 Department of Neurology, Henry Ford Health System, Detroit, MI USA; 7 Department of Biochemistry, Microbiology, and Immunology, Wayne State University, Detroit, MI USA

## Abstract

Previously, we reported that Zika virus (ZIKV) causes ocular complications such as chorioretinal atrophy, by infecting cells lining the blood-retinal barrier, including the retinal pigment epithelium (RPE). To understand the molecular basis of ZIKV-induced retinal pathology, we performed a meta-analysis of transcriptome profiles of ZIKV-infected human primary RPE and other cell types infected with either ZIKV or other related flaviviruses (Japanese encephalitis, West Nile, and Dengue). This led to identification of a unique ZIKV infection signature comprising 43 genes (35 upregulated and 8 downregulated). The major biological processes perturbed include SH3/SH2 adaptor activity, lipid and ceramide metabolism, and embryonic organ development. Further, a comparative analysis of some differentially regulated genes (ABCG1, SH2B3, SIX4, and TNFSF13B) revealed that ZIKV induced their expression relatively more than dengue virus did in RPE. Importantly, the pharmacological inhibition of ABCG1, a membrane transporter of cholesterol, resulted in reduced ZIKV infectivity. Interestingly, the ZIKV infection signature revealed the downregulation of ALDH5A1 and CHML, genes implicated in neurological (cognitive impairment, expressive language deficit, and mild ataxia) and ophthalmic (choroideremia) disorders, respectively. Collectively, our study revealed that ZIKV induces differential gene expression in RPE cells, and the identified genes/pathways (e.g., ABCG1) could potentially contribute to ZIKV-associated ocular pathologies.

## Introduction

The emergence of Zika virus (ZIKV) in both endemic and non-endemic regions of the world has been accompanied by an unprecedented rise in the spectra of ZIKV-associated diseases^1,2^. It is increasingly clear that ZIKV infection has broad implications beyond microcephaly, because infants born with congenital ZIKV have pathology in their eyes, ears and limbs^3–5^. Consequently, there has been significant interest in understanding the pathogenesis of ZIKV in various diseases. Because of clinical studies linking ZIKV to ocular abnormalities, primarily in the retina of infants and adults (uveitis)^6^, and our laboratory interests in innate retinal defense to microbial infections, we initiated host-pathogen interaction studies of ZIKV in the eye. In our recent study^7^, we reported for the first time that (1) ZIKV causes retinal lesions in mouse eyes with a clinical presentation [i.e., chorioretinal atrophy with retinal pigment epithelium (RPE) mottling] resembling some of the features of ZIKV-associated ocular pathology described in humans; (2) cells lining the blood-retinal barrier (BRB), the retinal vascular endothelium and RPE were permissive to ZIKV replication and expressed receptors for its entry; and (3) ZIKV induces retinal cell death and evokes innate retinal inflammatory and antiviral responses both *in vitro* and *in vivo*. These findings led us to postulate that being a blood-borne pathogen, ZIKV must overcome the BRB to gain access to the eye and cause retinal abnormalities, a key manifestation of ZIKV infection in the eye^6^. Moreover, three recent studies also support our findings by demonstrating the permissive nature of retinal endothelium and RPE to ZIKV infection^8–10^.

Since RPE constitutes the outer BRB, the specific contribution of RPE in ZIKV-induced retinal pathology such as chorioretinal atrophy remains to be elucidated. Similarly, upon infection, how ZIKV modulates innate antiviral responses and cell signaling in RPE for its own survival (replication) is currently unknown. Because ZIKV is closely related to other members of the Flaviviridae family such as dengue virus (DENV), West Nile virus (WNV), and Japanese encephalitis virus (JEV), an important question is why only ZIKV, but not other pathogenic flaviviruses, causes congenital diseases and associated complications^11^. We hypothesized that ZIKV might have a specific infection signature that distinguishes it from other flaviviruses. In this study, we generated a transcriptome profile of ZIKV-infected human primary RPE cells and performed a meta-analysis^12^ of transcriptome profiles of cells infected with other flaviviruses to gain insights in understanding the molecular mechanisms of ZIKV pathogenesis. Given the non-availability of vaccines against ZIKV, the results of this study will lead to the identification of novel pathways and/or drug targets to prevent ZIKV infection and its associated ocular complications.

## Results

### Meta-analysis scheme to identify ZIKV infection signature

**Figure 1:**
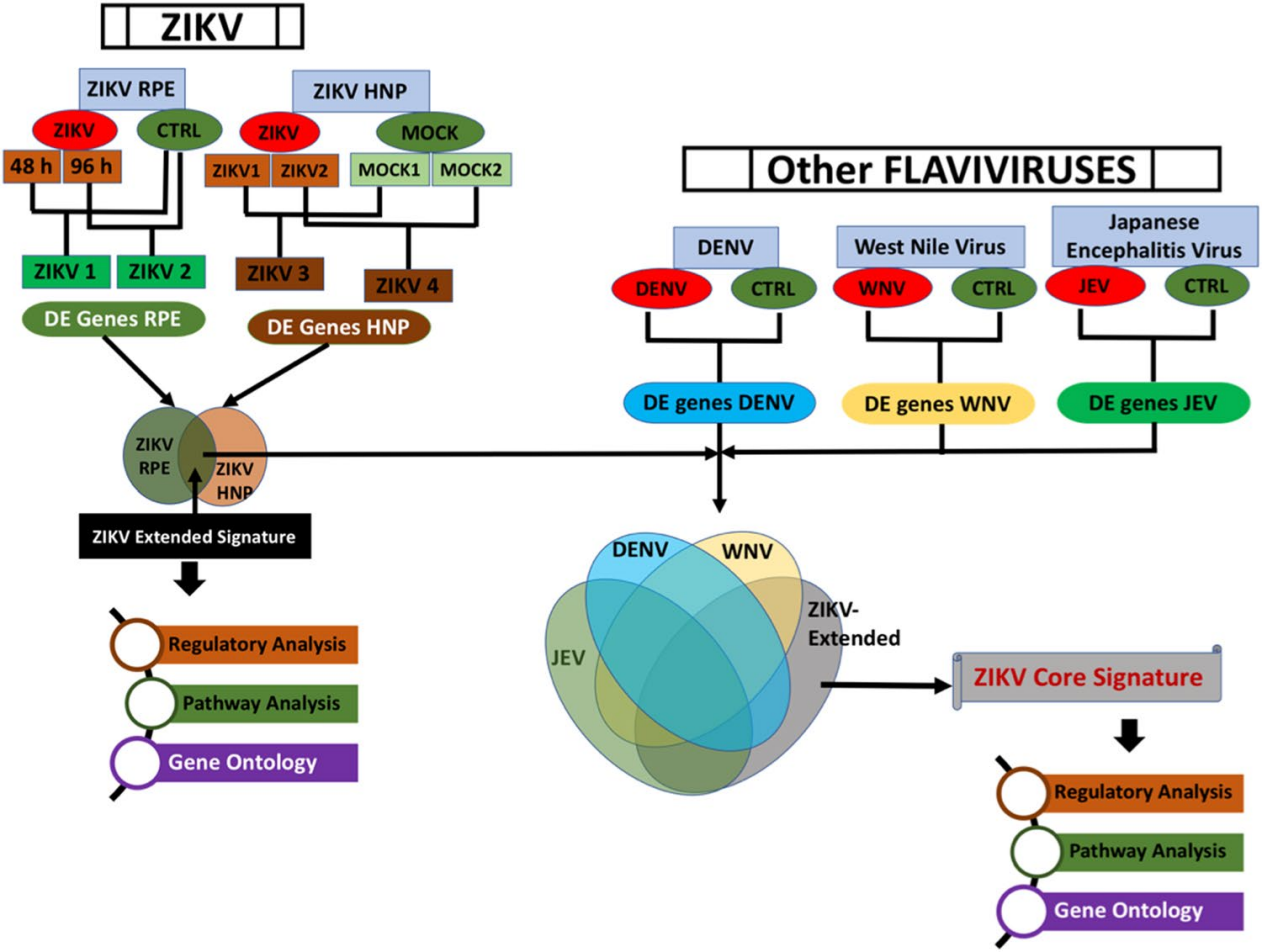
Overview of analytical meta-analysis approach to understand the molecular mechanism of ZIKV Infection. The comparative analysis of transcriptome alterations due to ZIKV infection in different cell types were performed to identify a meta-signature of ZIKV infection (left top). Furthermore, to identify the core set of genes unique to ZIKV infection, we also performed a comparative analysis of the ZIKV transcriptome with transcriptomes of DENV, WNV, and JEV (right middle). The genes only in the ZIKV extended set and not the other transcriptome comprise the ZIKV-core signature (middle right). Both the ZIKV extended and core-signature genes were evaluated by regulatory pathway and gene ontology analysis (bottom).

To identify key genes associated with ZIKV pathogenesis, we performed a comparative analysis of our ZIKV study with publicly available ZIKV studies and studies from other ZIKV related flaviviruses, such as JEV, WNV, and DENV. We performed transcriptome analysis of ZIKV-infected human primary RPE cells using ultra-sensitive RNA sequencing. The publicly available transcriptome data of ZIKV-infected neural progenitor cells and those infected with JEV, WNV, and DENV were obtained from the GEO database (https://www.ncbi.nlm.nih.gov/geo/) (Table 1) and normalized and analyzed using a uniform approach. The overview of the approach is shown in Fig. 1.

**Table 1:** List of transcriptome datasets used in current study.

Dataset	GEO ID	Platform	PMID
Zika virus	GSE78711	RNASEQ	26952870
Zika virus	GSE80434	RNASEQ	27580721
Dengue virus	GSE80434	RNASEQ	27580721
West Nile Virus	GSE30719	Affymetrix	22509103
Japanese encephalitis virus	GSE57330	Affymetrix	NA

### Principal component analysis (PCA) suggests that mock treated and ZIKV-infected RPE are transcriptionally different

**Figure 2:**
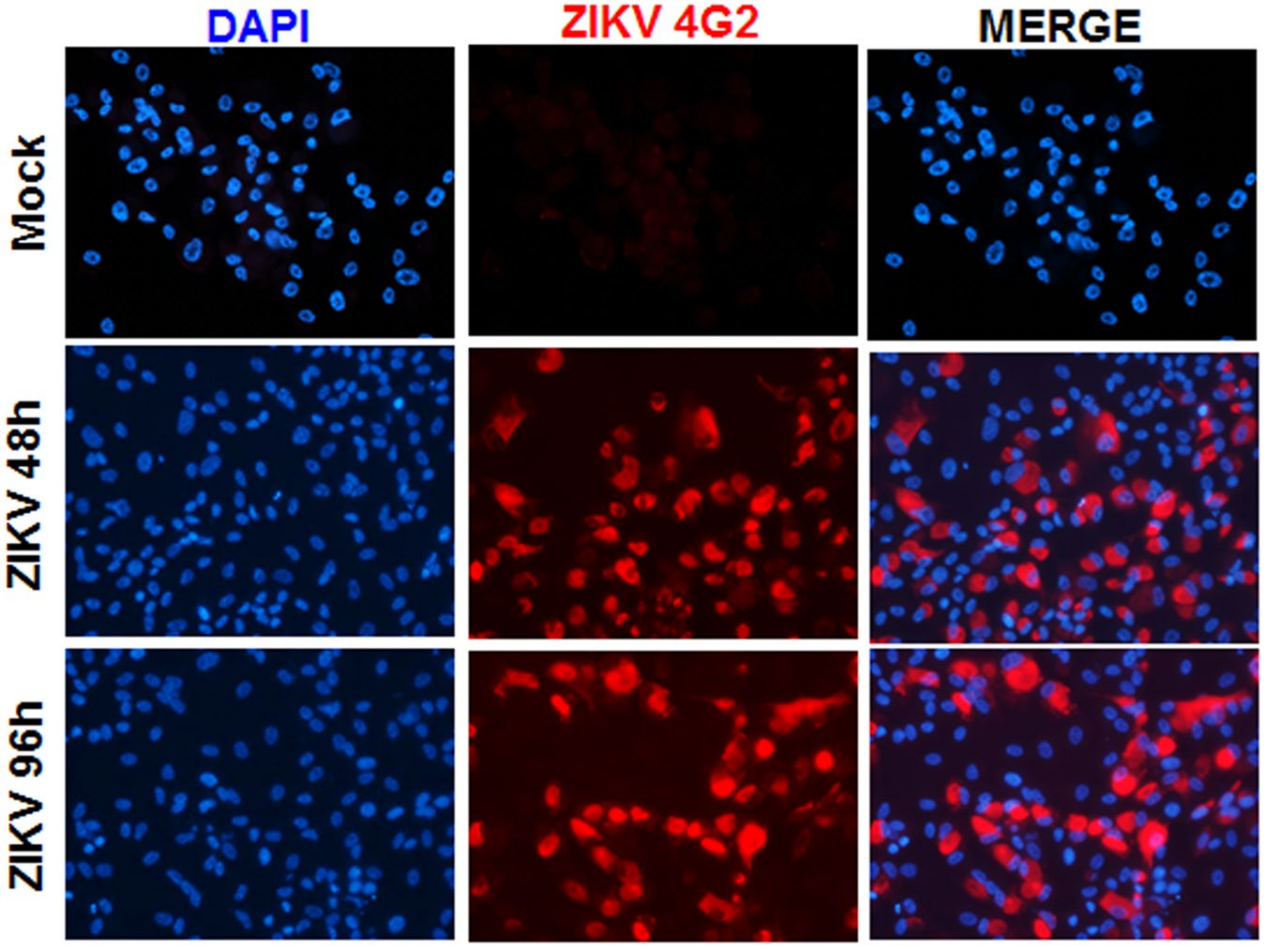
ZIKV PRVABC59 permissively infects human primary RPE cells. Human primary RPE cells were challenged with ZIKV (strain PRVABC59, PR 2015, MOI of 1) for the indicated time points; uninfected cells served as control (Mock). Control and ZIKV-infected cells were subjected to immunostaining for anti-flavivirus group antigen 4G2, and representative images show the presence of ZIKV (red) and DAPI (blue, a nuclear stain). Image magnifications 20X.

**Figure 3:**
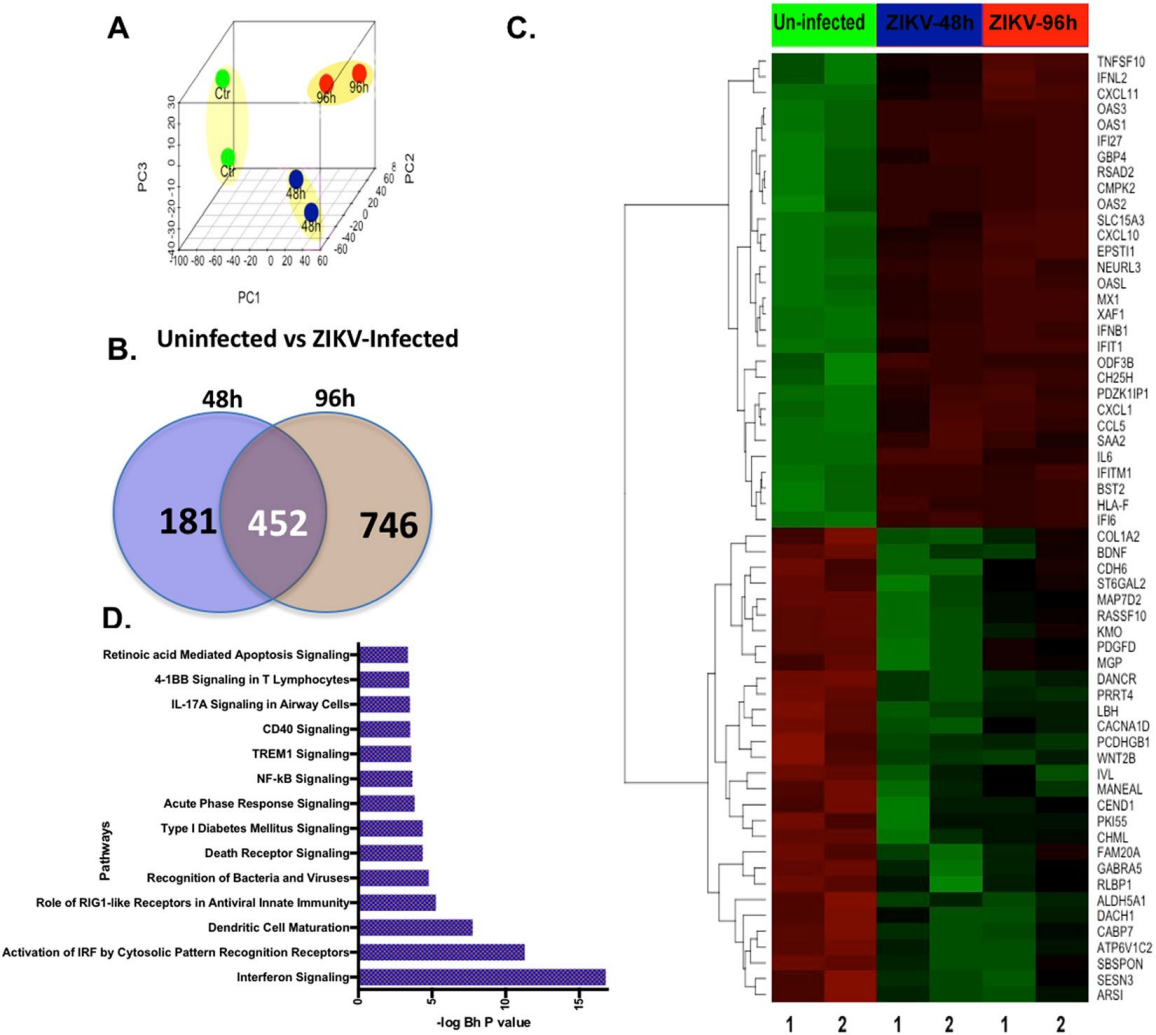
Transcriptome landscape of alterations in human primary RPE cells after ZIKV infection. (**A**) Unsupervised analysis of transcriptome profile of uninfected (Ctr), and ZIKV virus-infected primary RPE cells at 48 and 96 hrs post-infection, (**B**) Venn diagram depicting comparison of ZIKV-altered genes at 48 h and 96 h post-infection, **(C)** Heatmap of top altered genes due to ZIKV infection, and (**D**) Pathway analysis on genes significantly upregulated (>2 fold) in ZIKV-infected RPE cells. In the heatmap, columns represent the samples and rows represent the genes. Gene expression is shown with pseudocolor scale (−3 to 3) with red denoting high expression level and green denoting low expression level of a gene.

Transcriptome profiling was performed on uninfected control and ZIKV-infected (48 and 96 hrs) human primary RPE cells (Fig. 2) using ultra-sensitive robust paired-end RNA sequencing. After preprocessing and normalization of RNA sequencing data, we performed unsupervised analysis using PCA to determine the relationship between different groups as well as samples within each group. The PCA demonstrated that infection is the primary cause of transcriptional alteration, as shown along primary component (PC) 1 (Fig. 3A). The samples after 48 h and 96 h post infection also depicted slightly different transcriptional profiles, as shown along PC2. Biological replicates from infected and uninfected groups clustered together, indicating their similar transcriptome profile.

### Cell adhesion, interferon response, and neurogenesis genes are dysregulated due to ZIKV infection

The comparison of ZIKV-infected and uninfected samples at 48 h and 96 h identified 633 and 1,198 significantly differentially expressed genes respectively, with a P value < 0.01 and at least a 2-fold change, with 452 genes being commonly associated with ZIKV infection at both time points (Fig. 3B). Among these common genes, 414 were significantly upregulated, whereas 38 were downregulated (Table S1). Top selected ZIKV infection-associated genes from RPE cells are shown in a heatmap that includes multiple immune response genes, such as CCL5, CXCL11, CXCL1, IFNB1, IL6 and TNFSF10 (Fig. 3C). Further pathway enrichment analysis of ZIKV-upregulated genes depicted significant activation of antiviral response pathways including activation of “interferon signaling,” “Role of RIG I-like receptors in antiviral innate immunity,” “Recognition of bacteria and viruses,” “TREM1 signaling” and “CD40 signaling” (Fig. 3D).

### ZIKV infection in RPE cells specifically downregulates EIF2 signaling and mTOR signaling pathways in RPE cells

**Figure 4:**
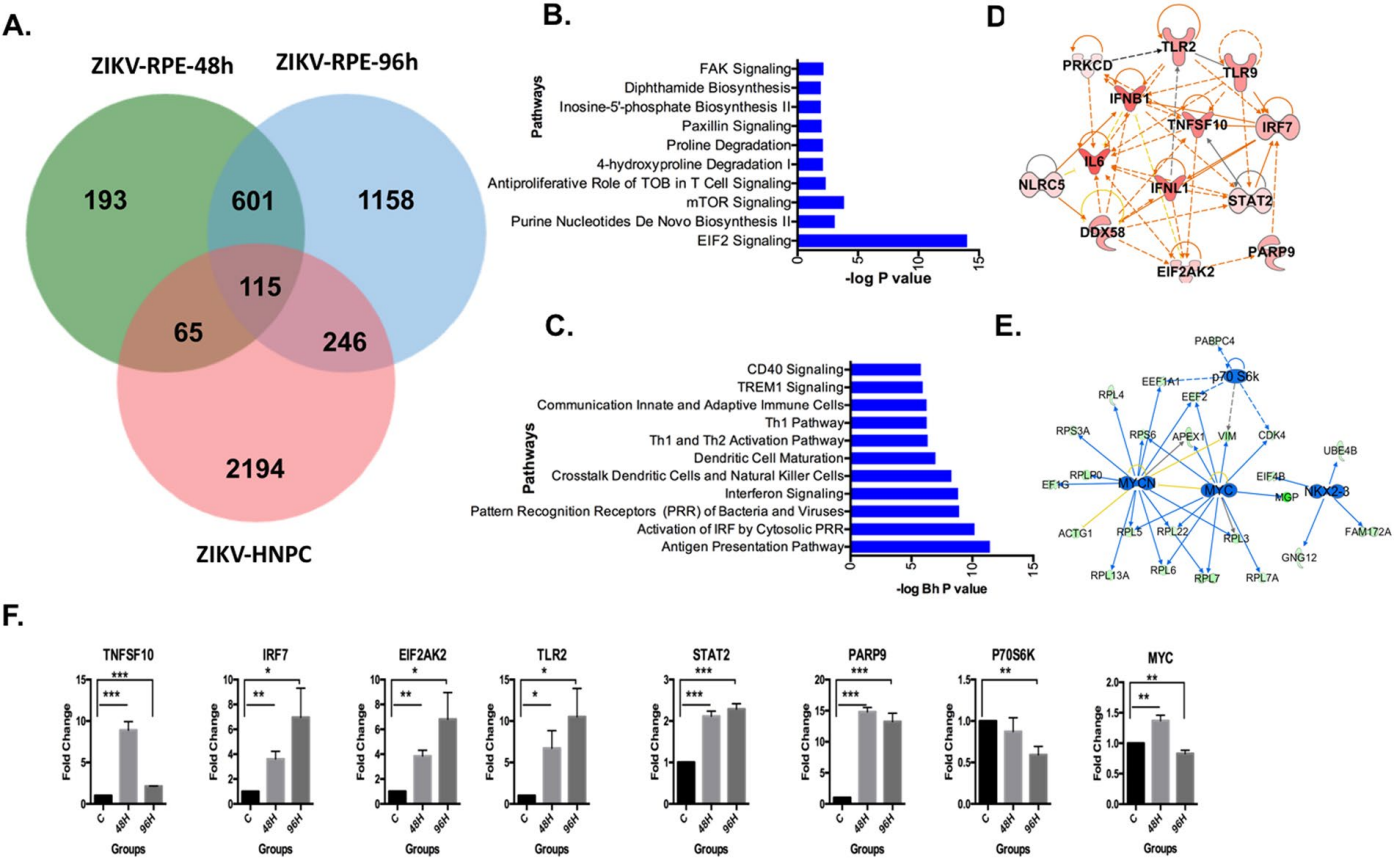
Primary retinal cell-specific ZIKV infection transcriptome signature. (**A**) Venn diagram comparing differentially expressed genes due to ZIKV infection of human primary RPE cells at 48 and 96 hrs and human neural progenitors cells (hNPC), (**B**) Pathways down-regulated due to ZIKV infection in RPE cells, (**C**) Pathways up-regulated due to ZIKV infection in RPE cells and key regulators that are activated (**D**) and inhibited (**E**) by ZIKV infection in RPE cells. The significance of effect on pathways was calculated using the Fisher exact test and shown as −log (P value) along the x-axis. The activated and inhibited key regulators are shown with orange and blue colors respectively. (**F**) Validation of key regulators altered by ZIKV infection in primary RPE cells. Uninfected control and ZIKV-infected human primary RPE cells were subjected to qRT-PCR analysis. Graphs represent the statistical analyses of relative mRNA levels after normalization to β-actin levels. Data shown here are mean ± SEM (standard error of the mean) from three independent experiments. *P < 0.09, **P < 0.05, and ***P < 0.01.

Next, we compared the transcriptome profiles of ZIKV-infected RPE with ZIKV-infected human neural progenitor cells (hNPCs). This led to the identification of a signature comprising 601 genes (484 upregulated and 117 downregulated) that were specifically dysregulated in ZIKV-infected RPE cells (Fig. 4A). These data suggest that ZIKV infection in retinal cells alters a significantly different set of genes compared to hNPCs. The gene ontology (GO) analysis on ZIKV-induced RPE-specific downregulated genes revealed a significant enrichment in biological processes linked to protein synthesis and translation, and nucleotide and amino acid metabolism (Fig. S1). A similar analysis on ZIKV-regulated genes in RPE revealed a significant enrichment in GO categories linked to innate antiviral defense and inflammatory responses comprising Type I and II interferon signaling, TLR signaling, MDA-5 signaling, and JAK-STAT cascade signaling (Fig. S2).

Further pathway analysis of genes that are specifically downregulated only in ZIKV-infected RPE cells revealed significant enrichment in EIF2 signaling, antiproliferative role of TOB in T cell signaling, mTOR signaling, paxillin signaling and FAK signaling pathways (Fig. 4B). On the other hand, upregulated genes were significantly enriched in immune response and inflammation pathways including “Antigen presentation pathway,” “Role of pattern recognition receptors in recognition of bacteria and viruses,” “Interferon signaling,” “TREM1 signaling” and “CD40 signaling” (Fig. 4C).

### Systems biology analysis identified inhibition of expression of MYC/MYCN and NKX-2 transcriptional regulators in RPE cells

To gain further insight into system-level perturbations and to identify key molecules associated with ZIKV Infection in RPE cells, we performed a systems biology-oriented analysis on the genes that are differentially expressed in RPE cells after ZIKV infection. The regulatory analysis revealed strong activation of immune and inflammatory responses, mediated through activation of TNFSF10, IFNB1, IL6, STAT2, TLR2, DDX54 (RIG-I), NLRC5, and IRF7, indicating that the RPE response to ZIKA infection is dominated by genes involved in inducing cytokines, chemokines, and type I interferons (Fig. 4D). On the other hand, ZIKV infection was found to inhibit the expression of MYC, MYCN and NKX2.3 transcription factors, which play roles in cell cycle, proliferation and cellular development (Fig. 4E). To validate these systems biology results, we confirmed the expression profile of significantly activated or inhibited key regulators using qRT-PCR. Our data showed a strong correlation between systems biology prediction and qRT-PCR validation of upregulated (e.g., TLR2, STAT2, TNFSF10, EIF2AK2, and PARP9) and downregulated (e.g., MYC and P70S6K) key regulators in ZIKV-infected primary human RPE cells (Fig. 4F).

### Meta-signature associated with ZIKV infection across other cell types

**Figure 5:**
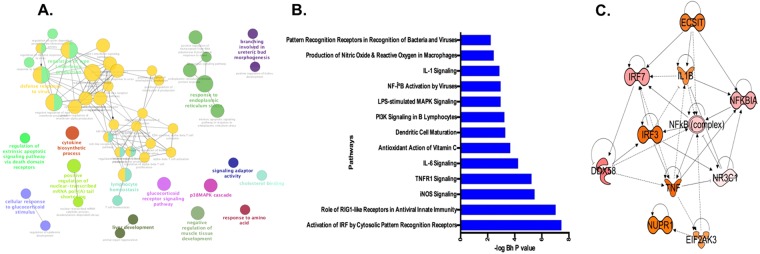
Transcriptional meta-signature associated with ZIKV infection. (**A**) Gene ontology clusters associated with ZIKV meta-signature. Dysregulated genes of multiple biological processes related to defense response against viruses and the type I interferon response. (**B**) Pathways are significantly dysregulated by ZIKV infection in multiple cell types. The significance of effect on pathways was estimated by one-tailed Fisher exact test. (**C**) Key transcriptional regulators significantly activated in ZIKV meta-signature.

The comparative analysis of ZIKV-infected RPE and hNPC signatures identified 115 common genes that were dysregulated consistently across both cell types (termed herein, ZIKV Meta-signature). Out of 115 genes, 99 were upregulated, and 16 were downregulated (Fig. 4A, central part). Most of the commonly dysregulated genes are enriched in biological processes significantly (P value < 0.05) linked to defense responses to viruses, e.g., type I interferon production, endoplasmic reticulum (ER) stress, cholesterol binding, p38 MAPKs, and apoptotic signaling (Fig. 5A). Further pathway analysis revealed significant activation of pathways linked to antiviral immune responses including “Role of RIG I-like receptors in antiviral innate immunity,” “TNFR1 signaling”, “IL-6 signaling”, “NF-kB activation by viruses” and “IL-1 signaling” (Fig. 5B). Upon systems biology analysis, these pathways indicated significant crosstalk with multiple immune and inflammation response-specific transcriptional regulators, such as DDX58 (RIG-I), TNF, IRFs, NFKB, and IL1B (Fig. 5C). Consistent with this, we have recently reported induced expression of some of these inflammatory mediators (TNFA, IL6, IL1B) and Interferons (IFNA1 and IFNB1) in ZIKV-infected primary RPE and retinal endothelial cells^7^, further validating systems biology prediction.

### Identification of 43 gene signature that is only perturbed by ZIKV Infection but not by other viruses

**Figure 6:**
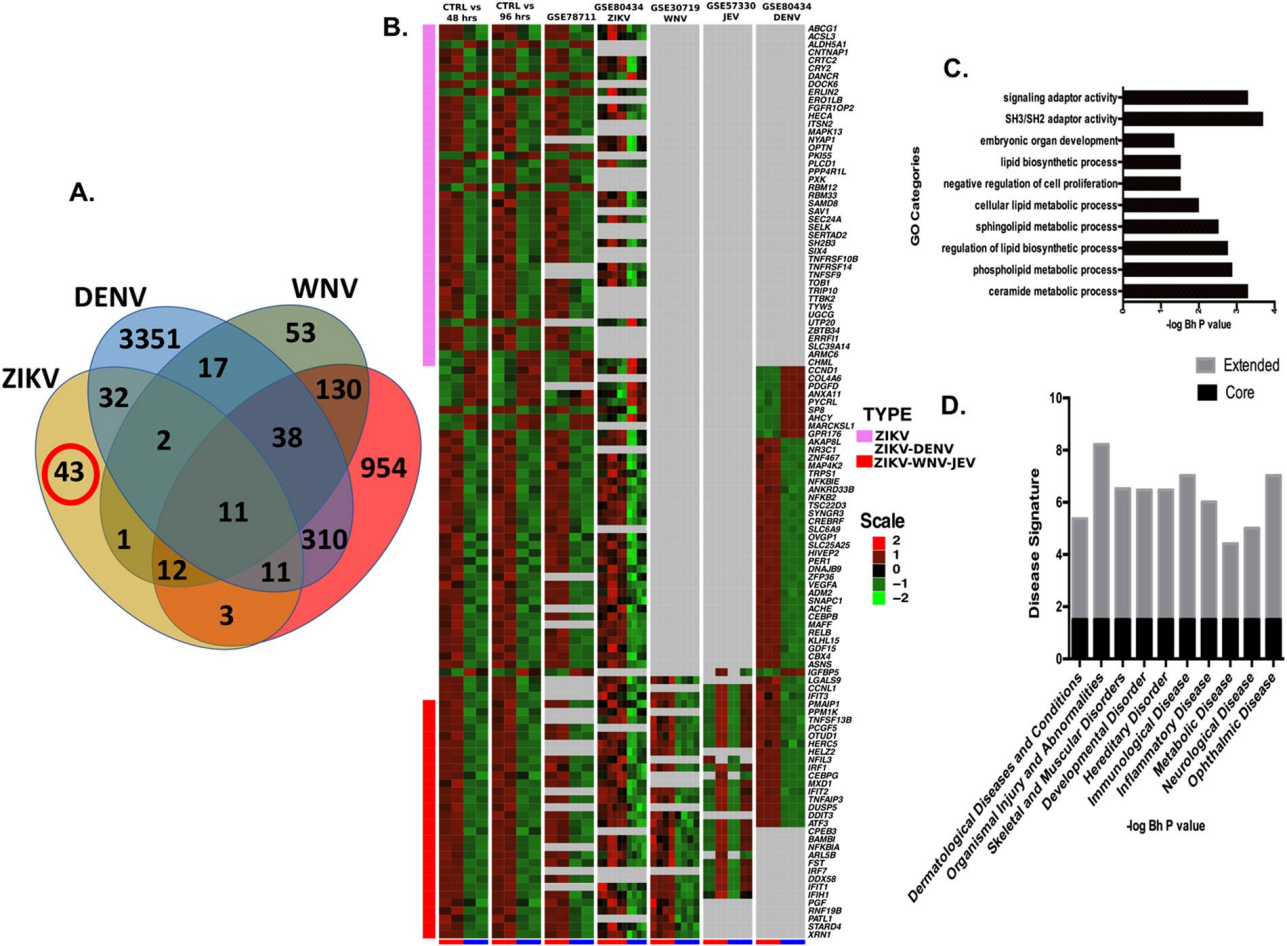
Identification of ZIKV infection-associated “Core transcriptional signature.” (**A**) Comparative analysis of ZIKV meta-signatures and other flavivirus transcriptomes using Venn diagram, (**B**) Heatmap of ZIKV core genes (pink sidebar) and genes overlapping with other neurological disease-causing viruses signatures (red sidebar). The middle part of the heatmap contains genes shared with other viruses. Columns represent different datasets used for identifying the ZIKV core signature (Blue at bottom: Normal and Red: Infected), and rows represent genes. Gene expression is shown with pseudocolor scale (−2 to 2) with red denoting high expression and green denoting low expression of a gene. (**C**) Gene ontology processes with enriched genes from the ZIKV core set. (**D**) Disease set with significant over-representation of the ZIKV core signature (Raw P-value < 0.01).

The meta-analysis of ZIKV signatures from different cell types (RPE versus hNPCs) was not sufficient to identify genes or pathways that are specifically linked to ZIKV infection or pathogenesis, because this analysis only revealed genes and pathways linked to general antiviral responses. To identify genes specifically dysregulated by ZIKV in addition to the generalized antiviral response, we performed a comparison of the ZIKV meta-signature with transcriptome signatures from other flaviviruses closely related to ZIKV, i.e., JEV, WNV, and DENV. Of the ZIKV meta-signature 115 genes (Fig. 4A), 72 genes were shared with other viruses, 34 genes were present only in ZIKV and DNV, 38 genes were shared between ZIKV, DENV, WNV, and JEV, whereas 14 genes were shared between ZIKV, WNV, and JEV. This comparative analysis identified 43 genes (designated as ZIKV core-signature hereafter) that were only dysregulated upon ZIKV infection but not by other viruses (Fig. 6A). This ZIKV core signature consisted of 35 upregulated and eight downregulated genes (Fig. 6B, Table 2).

**Table 2:** Core set of genes associated with ZIKV infection.

Count	Genes	CTR. vs 48 h	CTR. vs 96 h	GSE78711: ZIKV vs Control	GSE80434: ZIKV vs Control
1	ABCG1	3.155899	2.700842	0.843469	0.866306
2	ACSL3	1.013566	0.764678	0.800761	0.639925
3	CNTNAP1	2.281215	2.567525	0.664535	NA
4	CRTC2	0.704273	0.742567	0.833569	0.657603
5	CRY2	0.806888	1.005142	1.16703	0.931144
6	DDX58	5.33619604	4.25348802	1.11938785	NA
7	DOCK6	0.858289	1.074292	0.673731	NA
8	ERO1LB	1.667181	1.981114	0.882149	NA
9	FGFR1OP2	0.767468	0.906054	1.122912	0.671026
10	HECA	1.350149	1.939809	0.644875	0.726024
11	ITSN2	0.830577	1.054781	0.64226	NA
12	MAPK13	1.04260181	1.23386765	0.78187322	NA
13	NYAP1	1.91449	2.587236	NA	0.675337
14	OPTN	1.698412	1.217579	0.785475	0.667869
15	PLCD1	0.846584	0.982816	1.035717	0.858286
16	PPP4R1L	1.867549	1.963953	0.884417	NA
17	PXK	0.702051	1.108064	0.626402	NA
18	RBM33	0.751085	1.223488	0.69828	0.756224
19	SAMD8	0.837704	0.898175	0.673724	0.942943
20	SAV1	0.699783	0.868429	0.676048	NA
21	SEC. 24 A	0.669308	0.987638	1.08315	0.695181
22	SELK	1.107179	1.105049	0.712049	NA
23	SERTAD2	0.626871	0.871817	0.852495	NA
24	SH2B3	1.639506	1.72369	0.818069	0.653227
25	SIX4	0.766001	0.748324	0.835927	NA
26	SLC39A14	0.68938006	0.7869344	0.89250974	NA
27	TNFRSF10B	0.71554352	0.77481712	0.63166014	NA
28	TNFRSF14	1.20932852	1.5514799	NA	3.38441121
29	TNFSF9	4.006927	3.932652	NA	1.050304
30	TOB1	0.630485	0.762722	0.795475	0.814034
31	TRIP10	0.865456	1.269987	0.648967	NA
32	TTBK2	1.10199	1.799349	0.586193	NA
33	TYW5	0.805578	1.135104	0.761662	NA
34	UGCG	0.775482	1.390899	0.679913	NA
35	ZBTB34	0.995701	1.377278	0.783028	NA
36	ALDH5A1	−1.37322	−1.31132	−0.6077	NA
37	ARMC6	−0.7201926	−1.3836757	−1.0511958	NA
38	CHML	−1.2564619	−1.9774371	−0.86302	−0.8436419
39	DANCR	−2.20872	−2.59023	−1.01555	−1.03214
40	PKI55	−1.42331	−2.10882	−0.99145	NA
41	RBM12	−0.6996687	−0.7303744	−0.961712	NA
42	RBM12	−0.69967	−0.73037	−0.96171	NA
43	UTP20	−0.84553	−1.31002	NA	−0.7852

The table depicts log fold change in expression of genes because of ZIKV infection of primary RPE and neuronal progenitor cells.

Gene ontology enrichment analysis on ZIKV core signature genes revealed multiple biological processes related to SH3/SH2 adaptor activity, lipid and ceramide metabolism (P < 0.05) (Fig. 6C). Further disease set analysis on these genes identified significant enrichment in “Dermatological diseases, developmental disorders, immunological, metabolic, neurological and ocular disease sets (Fig. 6D). In addition, we also defined an extended ZIKV signature by including genes that were consistently dysregulated between ZIKV and other neurological disease-causing viruses, JEV and WNV (Table 2). The extended ZIKV signature consisted of 73 genes (39 core ZIKV-associated genes + 14 genes shared among ZIKV + JEV + WNV (Fig. 6B). The extended signature further enhanced the enrichment of ZIKV core signature genes in the above-identified diseases (Fig. 6B), supporting our hypothesis that the extended set of genes exacerbates the pathological effects of the core set of genes dysregulated by ZIKV.

### Determination of key molecules perturbed by ZIKV core signature

**Figure 7:**
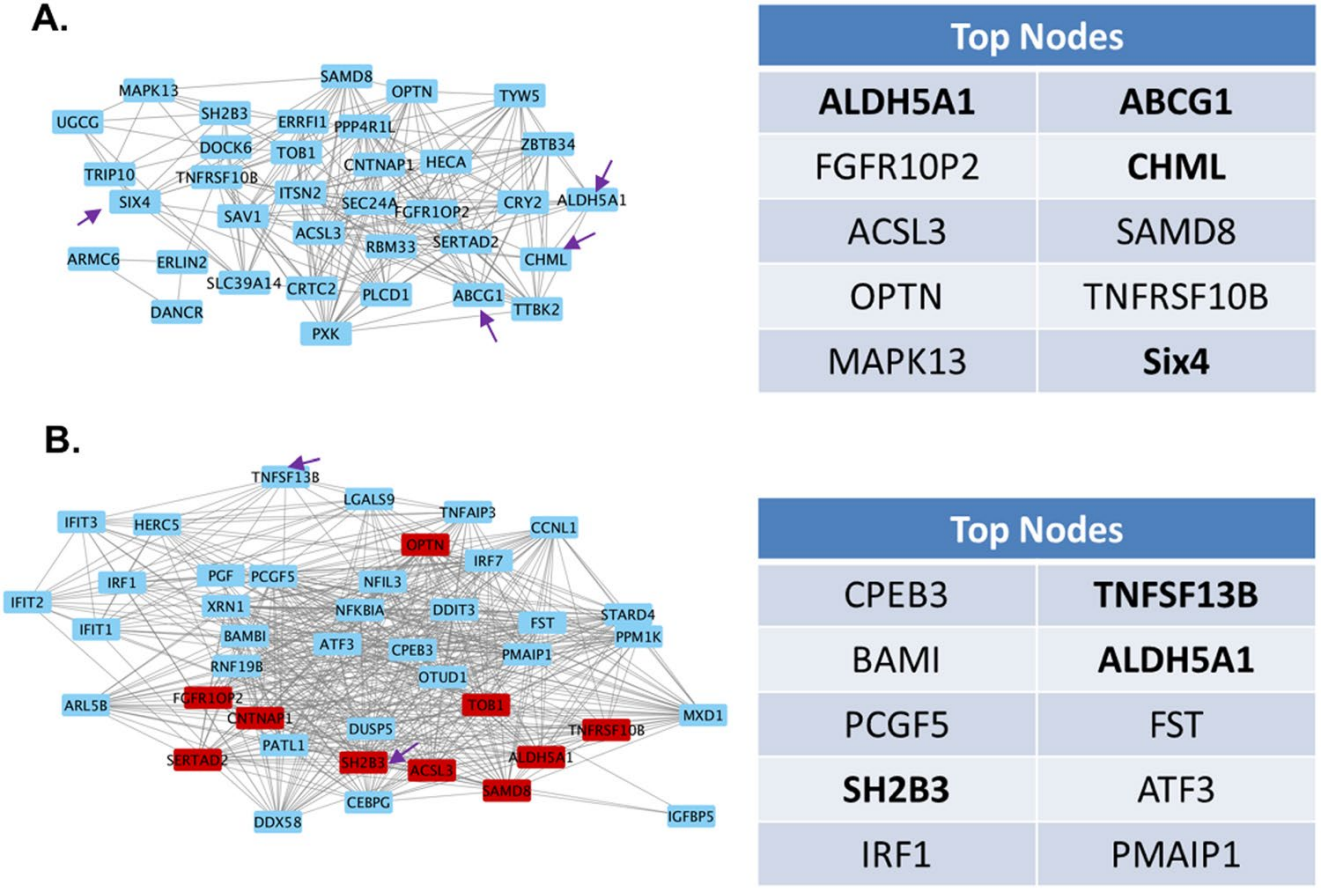
Identification of key regulators associated with ZIKV infection based on interactive network analysis. (**A**) Co-expression based interactive network of ZIKV-associated core signature. The topological analysis of the interactive network identified top 10 key regulators critical for the stability of the ZIKV infection-related interactive network. Each node represents a gene, and edges represent co-expression-based interactions. (**B**) Co-expression-based interactive network shows the interaction of top 10 key regulators from the ZIKV core network (red color) with the extended set of genes associated with ZIKV infection. The side panel lists the top 10 key regulators associated with ZIKV infection. CHML and ALDH5A1 are two key molecules significantly associated with stability of the ZIKV network prepared from the ZIKV core and the extended sets of genes.

Further interactive network analysis was performed on core ZIKV dysregulated genes to identify key regulators that are crucial for ZIKV pathophysiology. The interactive network was generated using the co-expression information of genes based on a Pearson correlation from the transcriptome profile of ZIKV and other viruses that cause neurological diseases. The network was constructed using highly correlative interactions of genes (correlation test P-value < 0.01). Since these networks are scale-free and undirected in nature, their topological properties reveal the impact of weak and strong connections measured based on “degree of centrality.” Our results showed significant enrichment of networks controlling immune, inflammation, and metabolic functions, mirroring the result of the GO analysis. Key nodes were isolated based on the degree of centrality ranking, and the top 10 key nodes included ALDH5A1, SIX4, ABCG1, TNFSF10B and CHML from the ZIKV core signature (Fig. 7A). To further explore the interaction between top key nodes associated with ZIKV infection and genes that are common between ZIKV and neurological disease-causing viruses, we performed extended network analysis. The topological analysis on the extended network identified SH2B adaptor protein 3 (SH2B3), FGFR10P2, ACSL3, OPTN, SAMD8, and TNFRFS13B as additional key molecules associated with ZIKV infection (Fig. 7B).

### Comparative analysis of ZIKV versus DENV in modulating the expression of meta-analysis predicted key genes in RPE

**Figure 8:**
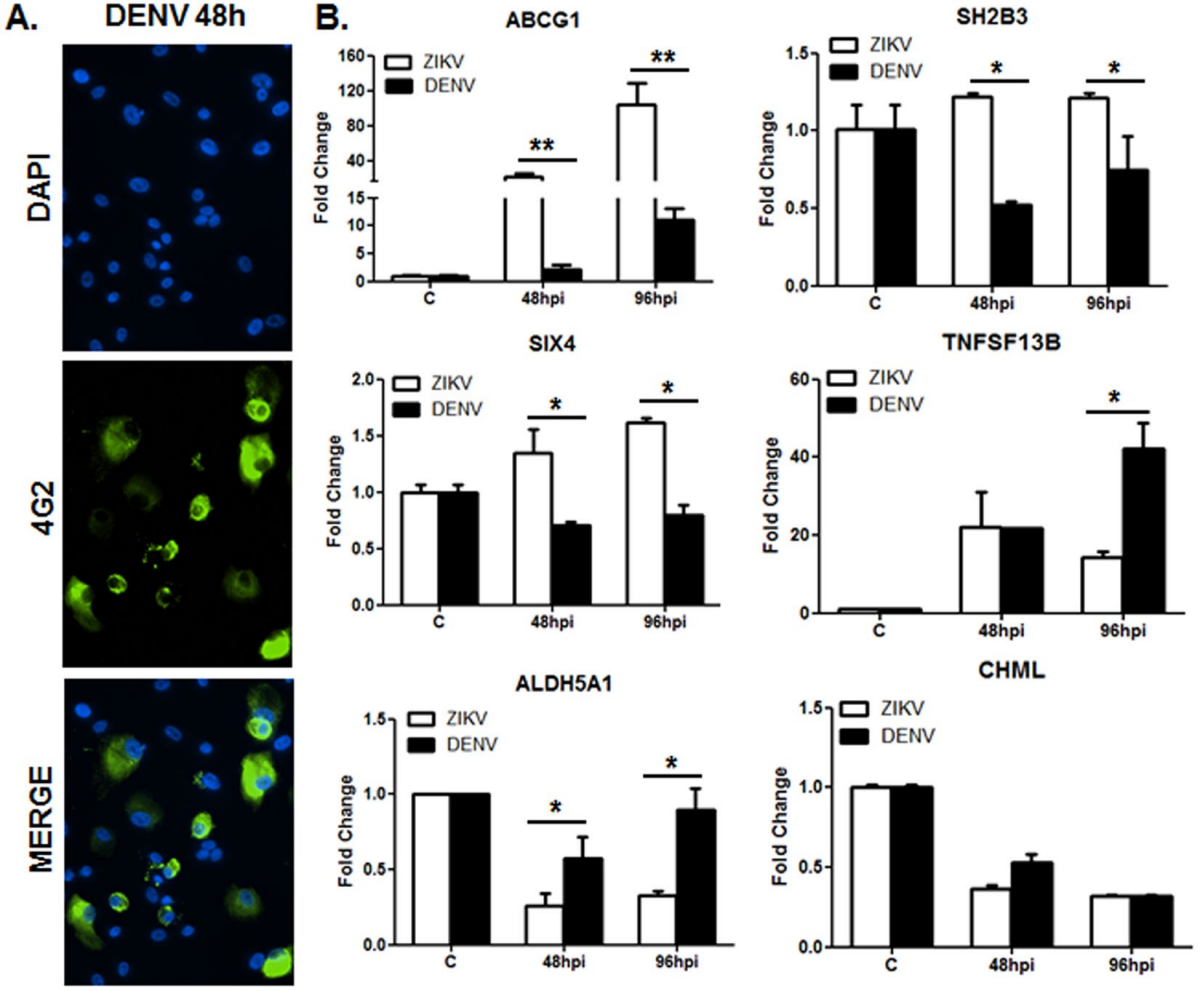
Comparative analysis of ZIKV and DENV in modulating meta-analysis-predicted genes in RPE cells. Human primary RPE cells were challenged with ZIKV or DENV, type 2, at MOI of 1 for the indicated time points; uninfected cells served as control. (**A**) DENV-infected cells were immunostained for anti-flavivirus group antigen 4G2 (image magnification 20X). (**B**) Expression of key regulator genes was assessed by qRT-PCR. Graphs show the statistical analyses of relative mRNA levels after normalization using β-actin. Results are expressed as mean ± S.D. of three independent experiments, *P < 0.05, **P < 0.005.

After meta-analysis and systems biology-led predictions of the ZIKV infection signature, we sought to determine whether other flaviviruses also modulate the expression of identified key molecules. First, we show that similar to ZIKV, human primary RPE cells are permissive to DENV (Fig. 8A). Second, the expression of select genes was determined in primary RPE challenged with either ZIKV or DENV. As predicted, ZIKV upregulated the expression of ABCG1, SH2B3, SIX4, and TNFSF13B in comparison to uninfected control cells. Similarly, DENV also induced the expression of ABCG1 and TNFSF13B but not SH2B3 and SIX4 in infected versus uninfected control cells. However, the comparative analysis showed significantly higher expression of ABCG1, SH2B3, SIX4 and TNFSF13B in ZIKV- versus DENV-infected RPE (Fig. 8B).

Among the genes predicted to be downregulated, qRT-PCR validation revealed that both ZIKV and DENV reduced the expression of ALDH5A1 and CHML in infected cells. While no significant difference was observed in downregulation of the CHML gene in ZIKV- versus DENV-infected cells, ALDH5A1 levels were drastically reduced by ZIKV compared to DENV at both time points 48 and 96 hrs post-viral challenge (Fig. 8B). These results indicate that ZIKV induces differential gene expression in retinal cells compared to other flaviviruses such as DENV.

### Functional role of ABCG1 in ZIKV pathogenesis

**Figure 9:**
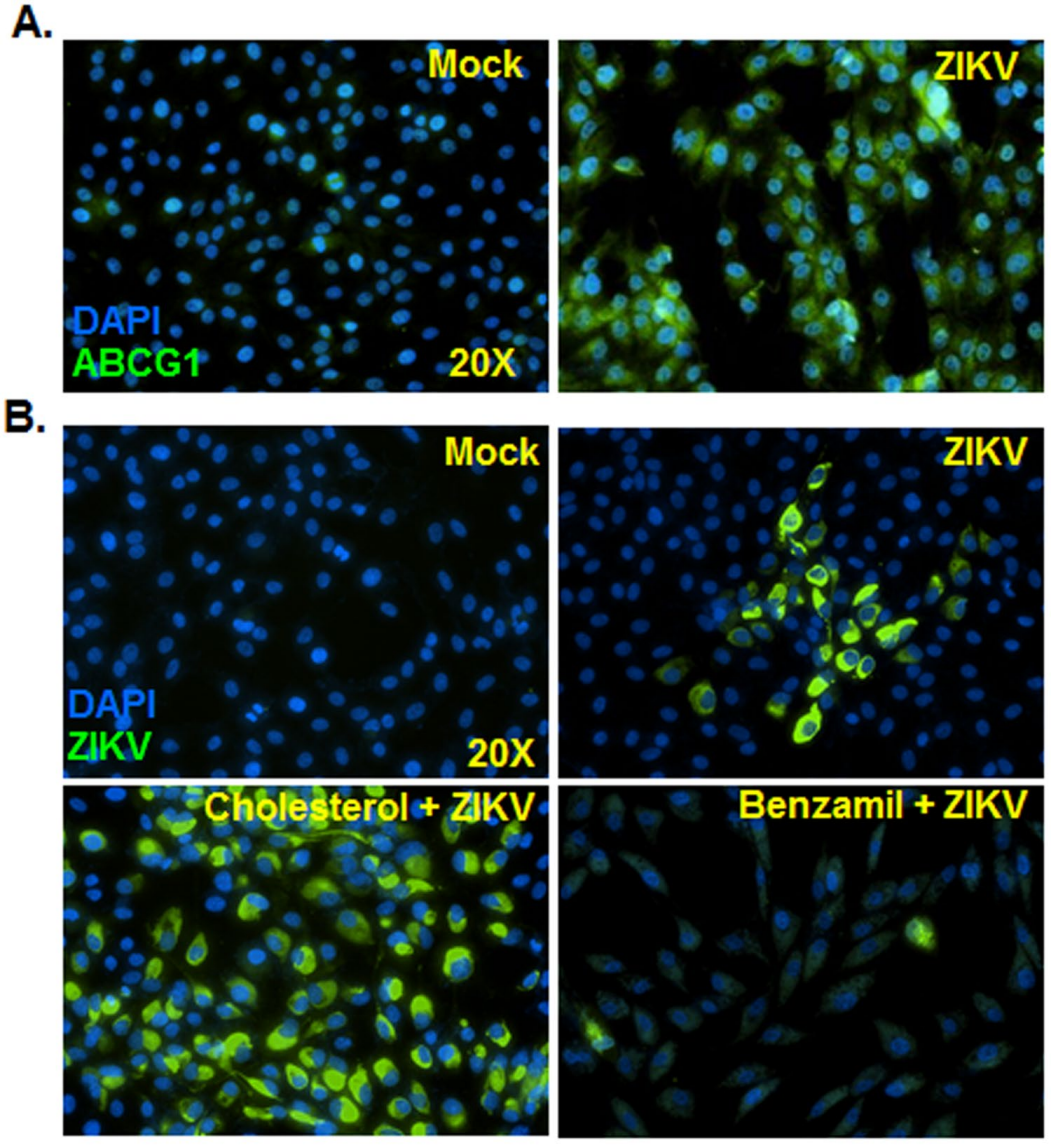
Pharmacological inhibition of ABCG1 attenuated ZIKV infectivity in RPE cells. Human primary RPE cells were challenged with ZIKV, and expression of ABCG1 was determined by immunostaining (**A**). In another set of experiments, RPE cells were pretreated with a pharmacological inhibitor of ABCG1, Benzamil (50 µM), or supplemented with cholesterol (10 µM) followed by infection with ZIKV (MOI of 1) for 48 h. ZIKV infectivity was assessed by anti-flavivirus group antigen 4G2 immunostaining (**B**). Results are representative of two independent experiments. Image magnifications 20X.

Among various predicted genes in our meta-analysis, we decided to assess the potential role of ABCG1 (a membrane transporter of cholesterol) because its expression has been demonstrated in the retina, including RPE cells^13–15^. In addition to mRNA (Fig. 8B), we confirmed ABCG1 expression at protein levels in ZIKV-infected RPE cells by immunostaining (Fig. 9A). Our results revealed that ZIKV-infected RPE expressed increased ABCG1, primarily as perinuclear/cytoplasmic punctae. To investigate the potential role of induced ABCG1 expression in RPE, we used Benzamil, an ABGC1 inhibitor^16,17^. Our data showed that pretreatment with Benzamil drastically attenuated ZIKV infectivity in RPE (Fig. 9B). In contrast, cholesterol supplementation increased ZIKV infectivity. Because ABCG1 is primarily involved in cholesterol efflux^18^, our results indicate the role of cholesterol homeostasis in ocular ZIKV pathogenesis.

## Discussion

The most recent outbreak of ZIKV in Brazil and other South American countries featured unexpectedly severe neurological manifestations that were not observed in prior ZIKV or other related flavivirus outbreaks^2,19^. This prompted the WHO to initially declare ZIKV as a public health emergency of international concern. Our interest in studying ZIKV pathogenesis emanated from clinical reports linking ZIKV with ocular complications such as uveitis^20,21^, acute maculopathy^22^, pigmentary retinopathy, and chorioretinal atrophy^23–26^. These clinical findings highlight that macular and chorioretinal disease can significantly impact visual outcomes in ZIKV-infected infants and adults. However, it is not yet clear whether congenital ocular complications are directly caused by ZIKV or are secondary to microcephaly^27^. To investigate whether ZIKV can directly affect the health of the retina, the primary target of ZIKV in the eye, we recently developed a mouse model of ZIKV-induced chorioretinal atrophy and demonstrated that cells lining the BRB and the retinal pigment epithelium (RPE) are highly permissive to ZIKV^7^.

Because RPE maintains the outer BRB and shields the neuroretina from blood-borne pathogens, RPE cell death as reported by our^7^ and other studies^8–10^ could create a portal for ZIKV entry to the eye from the fenestrated choroidal capillaries. To investigate the molecular mechanisms of ZIKV pathogenesis in RPE cells, here we performed transcriptome analysis of ZIKV-infected human primary RPE cells using a highly sensitive RNA sequencing approach. Consistent with the previous study of WNV-infected RPE cells^28^, our data also suggest the predominance of pathways regulating innate antiviral responses. These include interferon signaling, as indicated by upregulation of type-I IFNs, and the production of antiviral molecules, such as MX1, ISG15, OAS2, and CXCL10. Other pathways significantly upregulated in ZIKV-infected RPE were cytosolic pathogen recognition receptors like RIG-I, and activation of IRFs by cytosolic pattern recognition receptors and Toll-like receptors (TLRs), which play an essential role in recognition of viral RNAs, resulting in the modulation of IFN signaling. Indeed, our recent study showing ZIKV-induced antiviral innate responses in retinal endothelial cells and RPE, including induced expression of RIG-I and TLR3^7^, validates the transcriptome data obtained here. In addition to TLR3, unexpectedly, we observed the induction of TLR2 and TLR9, suggesting that these innate receptors might also be involved in recognition of ZIKV. The pathway analysis of genes that are specifically down-regulated in ZIKV-infected RPE showed significant enrichment in EIF2 signaling, mTOR signaling, paxillin signaling, and FAK signaling pathways, indicating the induction of cellular stress-related signals.

To identify the universal set of genes associated with ZIKV pathogenesis, we performed a comparative analysis of our transcriptome data with publicly available transcriptome data of other cell types infected by ZIKV. First, we compared our data with transcriptome data of ZIKV-infected human neural progenitor cells (hNPCs) from two other studies after uniform pre-processing and analysis. This led to the identification of 115 common genes that are dysregulated (99 upregulated, and 16 downregulated) consistently across both cell types (the ZIKV meta-signature). The most dysregulated pathways were linked to interferon production, ER stress, and defense response to viruses. This meta-analysis of ZIKV signatures from different cell types (RPE vs. neuronal) was unable to distinguish genes or pathways specifically linked to ZIKV pathogenesis from those of the common antiviral response. To further identify genes that are specifically dysregulated by ZIKV in addition to the generalized antiviral response, we performed a comparison of the ZIKV meta-signature of 115 genes with transcriptome signatures from DENV, JEV, and WNV. This meta-analysis led to the identification of a unique signature of 43 genes referred to as the ZIKV core signature, which is dysregulated upon ZIKV infection but not by the other flaviviruses tested. While the meta-analysis significantly reduces experimental efforts and assists in identifying the genes associated with a specific infection signature, the availability of only limited transcriptome datasets from similar cell types and/or time points (Table 1) for meta-analysis is a major limitation. However, in our experience from previous^29,30^ and current studies, meta-analysis after uniform normalization and preprocessing can provide a valid comparative analysis to identify most robust genes associated with pathophysiology. We normalized all the datasets and performed outlier analysis independently on all the studies to ensure that the studies are at the same normalized values and contain no outliers. Moreover, we have confirmed our meta-analysis findings using qRT-PCR analysis of identified key genes.

The gene ontology (GO) analysis of upregulated genes in the ZIKV core signature identified enrichment in multiple biological processes related to SH3/SH2 adaptor activity, lipid metabolism, and ceramide metabolism. This is consistent with the studies showing that WNV and DENV use ceramide differentially during their replication cycles^31^. Within the lipid metabolism gene set, one of the top genes in the ZIKV core signature was ABCG1, a membrane transporter critically involved in cholesterol efflux^18^. In addition to regulating lipid trafficking, ABCG1 modulates innate immune responses in macrophages^32,33^ and is abundantly expressed in both human and mouse retinal cells, including RPE^13^. Thus, based on our meta-analysis and the known expression of ABCG1 in RPE cells, we sought to determine its functional role in ocular ZIKV infection. In support our data show (1) validated ACBG1 expression both at mRNA and protein levels in ZIKV-infected RPE cells, and (2) that pharmacological inhibition of ACBG1^16,17^ resulted in reduced ZIKV infectivity, whereas cholesterol supplementation increased ZIKV infectivity in RPE cells. Viruses subvert cholesterol homeostasis using multiple mechanisms^34^. For example, DENV increases intracellular cholesterol levels by LDL uptake and promoting the activity of HMG-CoA reductase^35^, while herpes simplex virus 1 alters cholesterol trafficking^36^. Because host cell lipids and cholesterol play an essential role in various stages of viral replication, including entry, uncoating, genome replication, assembly, and release^37^, further studies should investigate how ZIKV infection of RPE alters chorioretinal cholesterol homeostasis^14,38^ and whether cholesterol transporters contribute to retinal pathology *in vivo,* using ABCG1^39,40^ or ABCA1^13^ (another cholesterol efflux transporter) knockout mice.

Another key regulator gene identified was SH2B3, originally characterized as LNK, a negative regulator of multiple cytokine signaling pathways, and which is associated with an increased risk of myocardial infarction^41^. Under inflammatory conditions, SH2B3/LNK counteracts leukocyte adhesion to endothelial cells by inhibiting VCAM-1 expression^42^. Endothelial cells lining the retinal blood vessels are the major regulatory interface for the trafficking of hematopoietic cells into the retina and provide an innate barrier to viral pathogens^43,44^, including ZIKV^7,9^. In response to inflammatory stimuli such as TNFA, activated endothelial cells highly express SH2B3^45^, which negatively regulates integrin signaling via inhibition of the integrin-linked kinase, thereby restricting endothelial cell adhesion and migration. SH2B3 also regulates integrins and cell motility in other cell types, such as platelets and megakaryocytes^46,47^. Our data showing induced expression of SH2B3 in ZIKV-infected retina/retinal cells indicates that ZIKV may employ SH2B3 to evade the immune system by attenuating the inflammatory response and infiltration of innate immune cells to the site of infection. The mechanism of SH2B3 modulation of the innate antiviral response to promote ZIKV replication needs further investigation, e.g., by inhibiting the SH2B3 activity or the use of *Lnk*^*−/*−^ knockout mice.

Among the key regulators downregulated by ZIKV was ALDH5A1, the gene encoding a mitochondrial NAD(+) -dependent succinic semialdehyde dehydrogenase (SSADH) enzyme. In humans, SSADH deficiency is a rare autosomal recessive metabolic disorder affecting γ-aminobutyric acid degradation and is characterized by developmental delay, cognitive impairment, expressive language deficit, and mild ataxia^48^. Epilepsy is present in about half of affected individuals^49^. Studies in *Aldh5a1*^*−/−*^ mice revealed that multiple metabolites associated with gamma-aminobutyric acid (GABA) metabolism (γ-hydroxybutyrate, succinic semialdehyde, D-2-hydroxyglutarate, gamma-hydroxybutyric acid (GHB), 4,5-dihydro hexanoate) and oxidative stress are significantly increased in multiple organs/tissues^50^. Myelin and lipid abnormalities in the cortex and hippocampus are also implicated in the pathophysiology of *Aldh5a1*^*−/−*^ mice^51^. Based on our findings demonstrating ZIKV-induced downregulation of the ALDH5A1 gene, we postulate that a reduction in ALDH5A1 enzyme activity would increase endogenous GHB and GABA levels, and therefore contribute to neurological manifestations of ZIKV infection such as microcephaly and Guillain-Barré syndrome in adults^52^. Further studies should address whether ZIKV indeed triggers SSADH and determine the underlying mechanisms. Another gene downregulated by ZIKV was CHML, which is linked to an ocular disorder called Choroideremia (CHM)^53^, a slowly progressing X-linked retinal disease characterized by degeneration of the choroid, the RPE, and the neural retina^54^. In both human and mouse eye, CHML and Rab escort-protein-1 (REP-1) are expressed throughout the retina in multiple cell types including RPE^54^. The CHML gene encodes REP-1, an essential player in the geranylgeranylation of *Ras*-related GTPases, which are Rab proteins involved in intracellular trafficking of vesicles in endocytic and exocytic pathways^55^. To determine whether ZIKV-infected RPE has impaired phagocytic function, we performed phagocytic uptake of fluorescent beads and observed no significant difference in infected versus uninfected control cells (data not shown). Silencing of REP-1 in RPE cells does not affect outer photoreceptor segment (POS) internalization but delays POS protein clearance and increases the secretion of inflammatory mediators^56^. The alteration of the activity of Rab proteins, including REP-1, in regulating endocytosis could be an important factor in the pathogenesis of diseases caused by intracellular pathogens^55^. Further studies are warranted to elucidate the function of the role of CHML in ZIKV and other flavivirus infections. The fact that ALDH5A1 and CHML, two key molecules significantly associated with the stability of the ZIKV network, are found in both ZIKV core and extended set of genes, indicates their importance in ZIKV-induced pathophysiology (Fig. 8).

In summary, to our knowledge, this study is the first to reveal the molecular signature of ZIKV infection, contributing to better understanding of ZIKV pathogenesis. By performing RNA sequencing on ZIKV infected human primary RPE cells and performing innovative comparative analysis of ZIKV transcriptome profiles with other flaviviruses, we identified the involvement of novel pathways unknown to play a crucial role in ZIKV pathogenesis. Moreover, in the wake of rapidly spreading ZIKV infection and lack of treatment options, the identified genes/pathways specifically perturbed by ZIKV infection could allow the rational design of therapeutic strategies to treat or prevent ZIKV infection and its complications.

## Methods

### Cell Culture and Viral infection

Human primary retinal pigmented epithelial (RPE) cells (Lonza, Walkersville, MD) were cultured in RtEGM^™^Bulletkit^®^ media as per manufacturer’s instruction (Lonza). Vero cells (ATCC CCL-81) and *Aedes albopictus* clone C6/36 cells (ATCC CRL-1660) were cultured in DMEM, and EMEM media supplemented with 10% FBS, 10 µg/ml L-glutamine, and 1% penicillin and streptomycin solution as per manufacturer’s recommendation. ZIKV Virus (ZIKV) strain PRVABC59, NR-50240, originally isolated from human blood in Puerto Rico in December 2015, and DENV type 2 strain NR12216 were obtained through BEI Resources, NIAID, NIH. This ZIKV strain is 97–100% genetically similar to the current ZIKV strain circulating in Brazil^57^. ZIKV and DENV were propagated in ATCC CCL-81 Vero cells and ATCC CRL-1660 C6/36 cells respectively, and titers were determined by plaque assay. RPE cells were infected with ZIKV and DENV at MOI of 1 for the indicated time points as described previously^7^. For ABCG1 functional studies, Pr. RPE cells were treated with a pharmacological inhibitor of ABCG1, Benzamil (50 µM) (Tocris Biosciences, Minneapolis, MN), and cholesterol-water soluble (10 µM) (Sigma Aldrich, St Louis, MO) 1 h before ZIKV challenge.

### Immunofluorescence Staining

For immunostaining procedures, primary RPE cells were cultured in a four-well chamber slide (Fisher Scientific, Rochester, NY) and infected with ZIKV and DENV at MOI of 1 and immunostained for anti-flavivirus group antigen 4G2 as described previously^7^. Briefly, cells were infected with ZIKV PRVABC59 and DENV NR12216 for the indicated time points. Following infection, ZIKV/DENV infected, and uninfected control cells were fixed overnight with 4% paraformaldehyde in PBS at 4 °C. Following rinse with PBS cells were incubated with primary mouse monoclonal antibody 4G2 (1:100) (Millipore, Billerica, MA, Cat # MAB10216) overnight at 4 °C. Following removal of the primary antibody, the cells were washed extensively with PBS and incubated for 1 h with anti-mouse Alexa Fluor 594-conjugated secondary antibody (1:200) at room temperature. Finally, the cells were extensively washed with PBS, and the slides were mounted in Vectashield anti-fade mounting medium containing DAPI (Vector Laboratories, Burlingame, CA) and visualized using fluorescence microscope (Nikon, Melville, NY).

### Transcriptome profiling of RPE cells using RNA sequencing

To understand the molecular mechanism of ZIKV infection, RNA derived from ZIKV-infected, and uninfected human primary RPE cells were subjected to next-generation sequencing to generate deep coverage RNA-Seq data. For each treatment group, sequencing was performed on at least two biological replicates. Sequencing libraries of Poly(A)-selected mRNA were generated from the double-stranded cDNA using the Illumina TruSeq kit, as per the manufacturer’s protocol. Library quality control was checked using the Agilent DNA High Sensitivity Chip and qRT-PCR. High-quality libraries were sequenced on an Illumina HiSeq. 2500. To achieve comprehensive coverage for each sample, we generated ~30 million paired-end reads from each sample.

### RNA sequencing data analysis from RPE cells

The raw sequencing data were processed to remove any adaptors, PCR primers and low-quality transcripts (removed leading or trailing bases with quality <3, removed parts of reads with average quality per 4 bases <15, filtered out reads shorter than 36 bases, kept only high quality paired reads) using the Trimmomatic program^58^. The quality of the reads with checked using FastQC program that provides a very comprehensive estimate of sample quality based on read quality, read length, GC content, uncalled based, the ratio of bases called, sequence duplication, adaptor and PCR primer contamination^59^. These high quality clean reads were aligned against human genome using Tophat2 algorithm that uses bowtie2 aligner (http://tophat.cbcb.umd.edu/)^60^. We used hg19 human genome assembly as a reference genome for alignment. Gene expression measurement was performed from aligned reads by counting the unique reads using the HTSeq-count function in HTSeq algorithm^61^. The features with low read counts were filtered out using counts per million approach. The filtered read count-based gene expression data was normalized using voom approach, which estimates the mean-variance relationship of the log-transformed gene count data and generates a precision weight for each gene expression^62^.

The differentially expressed genes were identified from normalized sequencing data using a linear model microarray analysis software package (Linear Models for Microarray Analysis (LIMMA))^63^. LIMMA estimates the differences between uninfected and infected samples by fitting a linear model and determining the significance of differences in gene expression by applying empirical Bayes moderated-t-statistics. The differentially expressed genes were identified based on absolute fold change and raw P value. Genes were considered significantly differentially expressed if the P value was <0.01 and absolute fold change >1.5. The unsupervised analysis was performed using principal component analysis (PCA) using prcomp library in R, which projects multivariate data objects onto a lower dimensional space while retaining as much of the original variance as possible.

### Meta-analysis of publicly available transcriptome studies to define the ZIKV meta-signature

To further establish the transcriptome alterations associated with ZIKV infection in different cell types and model systems, we performed a meta-analysis. For the meta-analysis, RNA sequencing or microarray data were obtained from public repositories, namely the GEO database (Table 1). The raw RNA sequencing data were preprocessed, aligned to the human genome (hg19), and unique reads were counted using Tophat2 and HTSeq count workflow as described previously^64^. The read count-based gene expression data were normalized on the basis of library complexity and gene variation using the R package EdgeR^65^. The normalized count data were compared among groups using a negative binomial model to identify differentially expressed genes. The differentially expressed genes were identified based on multiple tests corrected P value and fold change. Genes were considered significantly differentially expressed if the P-value was <5% false discovery rate (FDR) and absolute fold change >1.5. Heatmaps of differentially expressed genes were generated using heatmap.2 or complex heatmap packages in R^66^. In heatmaps, clustering of genes was performed using Hclust function in R using Euclidean distance, and complete linkage parameters on gene-wise scaled data. The scaled data was generated using “normalize” function of SOM package in R.

The meta-signature of ZIKV was generated by comparing the results of our study with other two published studies (Table 1). The comparison of differentially expressed genes among studies was performed based on gene symbols by generating Venn diagrams. The genes that were consistently differentially expressed among 75% of studies used in the meta-analysis were considered as the ZIKV meta-signature, and significantly associated with ZIKV.

### Identification of genes specifically perturbed by ZIKV Infection

To further identify molecular changes associated with only ZIKV infection, we also performed a comparative analysis of the ZIKV transcriptome with transcriptomes of other closely related flaviviruses such as DENV, WNV, and JEV. The raw transcriptome datasets for other viruses were obtained from the GEO database (Table 1). After quality control and preprocessing, transcriptome datasets were analyzed to identify genes dysregulated due to infection from different viruses. For DENV samples obtained from RNASeq data, the preprocessing and the identification of differentially expressed genes was performed similarly as discussed above. For analyzing WNV and JEV samples from Affymetrix, normalized microarray data was obtained from the GEO database using the GEOquery package^67^. The normalized data were generated using original Affymetrix CDF files. After rigorous quality control analysis and log (base 2) transformation of normalized data, the differential analysis was performed using LIMMA approach^63^. The mapping of Affymetrix probes to gene symbols and entrez IDs was performed using the biomaRt package in R^68^. The genes that were significantly dysregulated by different viral infections were identified by comparing uninfected and infected samples using LIMMA^63^. The genes with absolute fold change >2 and P-value < 0.05 FDR were considered significantly differentially expressed in this analysis. The comparison of the ZIKV meta-signature with transcriptome signatures from different viruses was performed on the basis of gene symbols using Venn diagram approach. The genes that were selectivity dysregulated in ZIKV-infected samples but not in other virus-infected samples were considered as the ZIKV core transcriptome signature.

### Gene ontology (GO) enrichment analysis

To identify the over-represented GO categories in host altered genes by ZIKV infection, we used the biological processes and molecular functions enrichment analysis available from the database for annotation, visualization and integrated discovery (DAVID)^69^. DAVID is an online implementation of the EASE software that produces a list of over-represented categories using jackknife iterative re-sampling of the Fisher exact probabilities. P values are assigned to each category based on enrichment. Smaller P values reflect increasing confidence in over-representation. The GO categories with P values < 0.05 and comprising at least 2 genes were considered significant. The GO enrichment analysis was performed both on meta-signature and core signatures of ZIKV infection to understand the key biological processes that are affected by ZIKV infection.

### Pathway and interactive network analysis

Ingenuity pathway analysis (IPA 8.0, Qiagen) was used to identify the pathways that are significantly affected by genes specifically associated with ZIKV infection. This software consists of functions, pathways and network models derived by systematically exploring the peer-reviewed the scientific literature. A detailed description of IPA analysis is available at the Ingenuity Systems’ website (http//www.ingenuity.com). It calculates a P value for each pathway according to the fit of users’ data to the IPA database using one-tailed Fisher exact test. The pathways with P values < 0.05 were considered significantly affected.

### Regulatory dysregulation analysis

Regulatory module analysis^70^ was used to identify the cascade of upstream transcriptional regulators that can explain the observed gene expression changes due to ZIKV infection. The analysis helps in identifying first which transcription regulators are significantly affected by the infection and then determining whether they are activated or inhibited. The activation or inhibition of transcriptional regulators was determined on the basis of overlap among our data with activation or inhibition signatures of key regulators. The significance of overlap was determined using the one-tailed Fisher exact test.

### Co-Expression network and master regulators perturbed by ZIKV infection

To define master regulators that are perturbed by genes associated with ZIKV infection, we performed an interactive network analysis. Co-expression-based interactive networks were developed from normalized expression matrix data from ZIKV studies (i.e., ZIKV-core signature) and an extended group of viruses (i.e., ZIKV, WNV, JEV) causing neurological diseases. For co-expression analysis, network analysis returns pairwise gene interactions, Pearson R values (t-static), P values and Bonferroni-adjusted P values. We restricted interactions building with a P value < 0.01 to identify high confidence hub genes that regulated the network. The topological network parameters were calculated using cytoNCA package in Cytoscape^71^. The topological analysis was performed on the network to identify nodes critical for network stability. We performed topological analysis using the degree of the node of centrality parameter, which is a standardized measure to calculate a number of edges connected to each node^72^.

### RNA extraction and qRT-PCR

Total RNA was extracted from mock-treated and ZIKV/DENV-infected RPE cells using RNeasy Mini kit (Qiagen, Valencia, CA), per the manufacturer’s instructions. An aliquot of the same RNA was used for RNAseq analysis and another aliquot for gene validation studies by qRT-PCR. For qRT-PCR, cDNA was synthesized using 1 µg of total RNA using a Maxima first strand cDNA synthesis kit, as per the manufacturer’s instructions (Thermo Scientific). The cDNA was amplified using gene-specific PCR primers. qRT-PCR was conducted in a StepOnePlus™ Real-Time PCR system (Applied Biosystems). Integrated DNA Technologies synthesized all the primers. The quantification of gene expression was determined via the comparative ΔΔCT method. Gene expression data in the test samples were normalized to the endogenous reference β-actin level and were reported as fold change relative to β-actin gene expression. All assays were performed in triplicate and repeated at least twice. The data are presented as the mean ± SD, and statistical analysis was performed using Student’s t-test or one-way ANOVA followed by post hoc test using GraphPad Prism 7.02 (La Jolla, CA).

## Electronic supplementary material

Supplementary Information
