# Supplementary material for: Determination of system level alterations in host transcriptome due to Zika virus (ZIKV) Infection in retinal pigment epithelium
Source: Sci Rep. 2018 Jul 25;8:11209. doi: 10.1038/s41598-018-29329-2 (PMC6060127; doi:10.1038/s41598-018-29329-2)
Supplement: Supplementary file 1 — Supplementary Information [file 41598_2018_29329_MOESM1_ESM.docx]

**Title: Determination of system level alterations in host transcriptome due to Zika virus (ZIKV) Infection in retinal pigment epithelium**

**Authors:** Pawan Kumar Singh^1 †^, Indu Khatri^2,3 †^, Alok Kumar Jha^2,3^, Carla D. Pretto^4^ , Katherine R. Spindler^4^, Vaithilingaraja Arumugaswami^5^, Shailendra Giri^6^, Ashok Kumar^1, 7*^ and Manoj K. Bhasin^2,3*,^

† First author/equal contribution

**Supplementary Figures:**

**Fig S1:** Gene Ontology enrichment analysis on RPE specific downregulated genes. The analysis depicted a significant enrichment in biological processes linked to protein synthesis and translation and nucleotide metabolism.


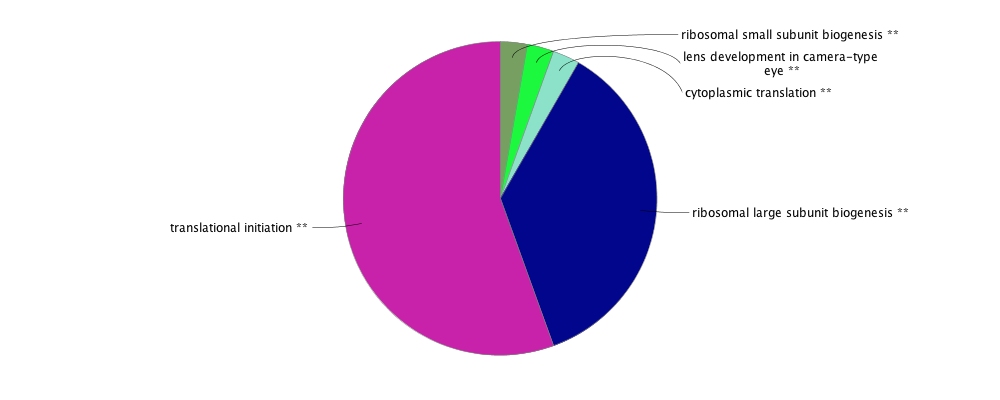


**Fig S2:** Gene Ontology enrichment analysis on RPE specific upregulated genes. The analysis depicted a significant enrichment in biological processes linked to GO categories linked Innate Immune and inflammatory response and Antiviral Type I and II interferon signaling.


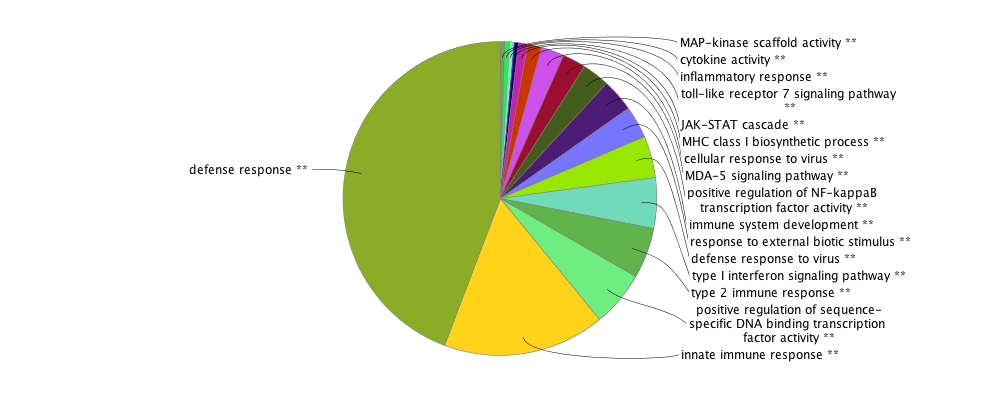


**Supplementary Table:**

**Table S1:** List of genes that are commonly altered at 48hrs and 96hrs post Zika infection to primary retinal cells. List contain genes that are significantly altered (Absolute Fold change > 2 and P value <.01) due to Zika infection in primary retinal cells.

| Genes | Description | **Log Fold Change** | |
| --- | --- | --- | --- |
|  |  | CTR-48 | CTR-96 |
| IVL | involucrin [Source:HGNC Symbol;Acc:HGNC:6187] | -3.301358692 | -3.702412623 |
| KMO | kynurenine 3-monooxygenase [Source:HGNC Symbol;Acc:HGNC:6381] | -2.378715478 | -4.633099443 |
| ATP6V1C2 | ATPase H+ transporting V1 subunit C2 [Source:HGNC Symbol;Acc:HGNC:18264] | -2.338938731 | -2.361543231 |
| CACNA1D | calcium voltage-gated channel subunit alpha1 D [Source:HGNC Symbol;Acc:HGNC:1391] | -2.246465532 | -3.547953208 |
| DANCR | differentiation antagonizing non-protein coding RNA [Source:HGNC Symbol;Acc:HGNC:28964] | -2.208716844 | -2.590233439 |
| PRRT4 | proline rich transmembrane protein 4 [Source:HGNC Symbol;Acc:HGNC:37280] | -1.959084307 | -2.221722406 |
| RASSF10 | Ras association domain family member 10 [Source:HGNC Symbol;Acc:HGNC:33984] | -1.837280959 | -3.702389286 |
| DACH1 | dachshund family transcription factor 1 [Source:HGNC Symbol;Acc:HGNC:2663] | -1.784002308 | -1.687349173 |
| CABP7 | calcium binding protein 7 [Source:HGNC Symbol;Acc:HGNC:20834] | -1.779666503 | -2.066684601 |
| FAM20A | FAM20A, golgi associated secretory pathway pseudokinase [Source:HGNC Symbol;Acc:HGNC:23015] | -1.773183794 | -3.210059765 |
| ST6GAL2 | ST6 beta-galactoside alpha-2,6-sialyltransferase 2 [Source:HGNC Symbol;Acc:HGNC:10861] | -1.737419676 | -4.155063025 |
| SBSPON | somatomedin B and thrombospondin type 1 domain containing [Source:HGNC Symbol;Acc:HGNC:30362] | -1.67298707 | -1.947684314 |
| MANEAL | mannosidase endo-alpha like [Source:HGNC Symbol;Acc:HGNC:26452] | -1.636160849 | -2.311114177 |
| PDGFD | platelet derived growth factor D [Source:HGNC Symbol;Acc:HGNC:30620] | -1.501818022 | -3.810577278 |
| PCDHGB1 | protocadherin gamma subfamily B, 1 [Source:HGNC Symbol;Acc:HGNC:8708] | -1.463151049 | -1.589429674 |
| WNT2B | Wnt family member 2B [Source:HGNC Symbol;Acc:HGNC:12781] | -1.461380596 | -1.596862785 |
| CEND1 | cell cycle exit and neuronal differentiation 1 [Source:HGNC Symbol;Acc:HGNC:24153] | -1.442457874 | -2.157694357 |
| ARSI | arylsulfatase family member I [Source:HGNC Symbol;Acc:HGNC:32521] | -1.435290109 | -1.440779671 |
| PKI55 |  | -1.423313551 | -2.108820117 |
| ALDH5A1 | aldehyde dehydrogenase 5 family member A1 [Source:HGNC Symbol;Acc:HGNC:408] | -1.373222806 | -1.311318355 |
| RLBP1 | retinaldehyde binding protein 1 [Source:HGNC Symbol;Acc:HGNC:10024] | -1.366348761 | -2.054292677 |
| GABRA5 | gamma-aminobutyric acid type A receptor alpha5 subunit [Source:HGNC Symbol;Acc:HGNC:4079] | -1.334000933 | -1.890706139 |
| NEXN | nexilin F-actin binding protein [Source:HGNC Symbol;Acc:HGNC:29557] | -1.328222391 | -1.186325535 |
| PCDHGA2 | protocadherin gamma subfamily A, 2 [Source:HGNC Symbol;Acc:HGNC:8700] | -1.326424197 | -1.011330406 |
| CDH6 | cadherin 6 [Source:HGNC Symbol;Acc:HGNC:1765] | -1.266922535 | -3.326042911 |
| SESN3 | sestrin 3 [Source:HGNC Symbol;Acc:HGNC:23060] | -1.258737322 | -1.401056008 |
| CHML | CHM like, Rab escort protein 2 [Source:HGNC Symbol;Acc:HGNC:1941] | -1.256461856 | -1.977437136 |
| LBH | limb bud and heart development [Source:HGNC Symbol;Acc:HGNC:29532] | -1.192016829 | -1.608831449 |
| MAP7D2 | MAP7 domain containing 2 [Source:HGNC Symbol;Acc:HGNC:25899] | -1.179457662 | -2.056604198 |
| LMCD1 | LIM and cysteine rich domains 1 [Source:HGNC Symbol;Acc:HGNC:6633] | -1.130343748 | -1.202577417 |
| MGP | matrix Gla protein [Source:HGNC Symbol;Acc:HGNC:7060] | -1.117972379 | -3.186522714 |
| HSD17B2 | hydroxysteroid 17-beta dehydrogenase 2 [Source:HGNC Symbol;Acc:HGNC:5211] | -1.078256473 | -1.480783242 |
| BDNF | brain derived neurotrophic factor [Source:HGNC Symbol;Acc:HGNC:1033] | -1.076107301 | -1.608697626 |
| COL1A2 | collagen type I alpha 2 chain [Source:HGNC Symbol;Acc:HGNC:2198] | -1.072138981 | -1.874717571 |
| SCD5 | stearoyl-CoA desaturase 5 [Source:HGNC Symbol;Acc:HGNC:21088] | -1.070408026 | -1.327094193 |
| PRADC1 | protease associated domain containing 1 [Source:HGNC Symbol;Acc:HGNC:16047] | -1.061965338 | -1.189177209 |
| POLR1E | RNA polymerase I subunit E [Source:HGNC Symbol;Acc:HGNC:17631] | -1.05316607 | -1.464636343 |
| AADAT | aminoadipate aminotransferase [Source:HGNC Symbol;Acc:HGNC:17929] | -1.02830455 | -1.323539305 |
| CACNB1 | calcium voltage-gated channel auxiliary subunit beta 1 [Source:HGNC Symbol;Acc:HGNC:1401] | 1.007324061 | 1.150301985 |
| NID1 | nidogen 1 [Source:HGNC Symbol;Acc:HGNC:7821] | 1.008490046 | 1.68378139 |
| NLGN4Y | neuroligin 4, Y-linked [Source:HGNC Symbol;Acc:HGNC:15529] | 1.012031843 | 1.360423157 |
| N4BP1 | NEDD4 binding protein 1 [Source:HGNC Symbol;Acc:HGNC:29850] | 1.016741188 | 1.13934315 |
| ABCC3 | ATP binding cassette subfamily C member 3 [Source:HGNC Symbol;Acc:HGNC:54] | 1.021149591 | 1.857228847 |
| VSIG10L | V-set and immunoglobulin domain containing 10 like [Source:HGNC Symbol;Acc:HGNC:27111] | 1.023065946 | 1.233660349 |
| HOOK2 | hook microtubule tethering protein 2 [Source:HGNC Symbol;Acc:HGNC:19885] | 1.02503028 | 1.310042729 |
| RRAS | related RAS viral (r-ras) oncogene homolog [Source:HGNC Symbol;Acc:HGNC:10447] | 1.028137301 | 1.124747509 |
| NFATC4 | nuclear factor of activated T-cells 4 [Source:HGNC Symbol;Acc:HGNC:7778] | 1.034837523 | 1.20921226 |
| VASN | vasorin [Source:HGNC Symbol;Acc:HGNC:18517] | 1.03613335 | 1.65366489 |
| MAPK13 | mitogen-activated protein kinase 13 [Source:HGNC Symbol;Acc:HGNC:6875] | 1.042601805 | 1.233867652 |
| IER3 | immediate early response 3 [Source:HGNC Symbol;Acc:HGNC:5392] | 1.048961565 | 2.146835204 |
| ARRDC2 | arrestin domain containing 2 [Source:HGNC Symbol;Acc:HGNC:25225] | 1.049673616 | 1.054557007 |
| IRF9 | interferon regulatory factor 9 [Source:HGNC Symbol;Acc:HGNC:6131] | 1.051896482 | 1.224163901 |
| MTMR9LP | myotubularin related protein 9-like, pseudogene [Source:HGNC Symbol;Acc:HGNC:27920] | 1.060779926 | 1.714018445 |
| COL7A1 | collagen type VII alpha 1 chain [Source:HGNC Symbol;Acc:HGNC:2214] | 1.065294405 | 1.222771263 |
| GDF15 | growth differentiation factor 15 [Source:HGNC Symbol;Acc:HGNC:30142] | 1.066748793 | 2.567507199 |
| TMEM63A | transmembrane protein 63A [Source:HGNC Symbol;Acc:HGNC:29118] | 1.068718649 | 1.37586544 |
| UBR4 | ubiquitin protein ligase E3 component n-recognin 4 [Source:HGNC Symbol;Acc:HGNC:30313] | 1.07155174 | 1.288258242 |
| TNC | tenascin C [Source:HGNC Symbol;Acc:HGNC:5318] | 1.091618797 | 1.060371152 |
| WTAP | Wilms tumor 1 associated protein [Source:HGNC Symbol;Acc:HGNC:16846] | 1.098091785 | 1.801048575 |
| TTBK2 | tau tubulin kinase 2 [Source:HGNC Symbol;Acc:HGNC:19141] | 1.101989887 | 1.799349467 |
| JAK3 | Janus kinase 3 [Source:HGNC Symbol;Acc:HGNC:6193] | 1.102584084 | 2.285650311 |
| VEGFA | vascular endothelial growth factor A [Source:HGNC Symbol;Acc:HGNC:12680] | 1.106738631 | 1.510491654 |
| SELK |  | 1.107179369 | 1.105049426 |
| ITPRIP | inositol 1,4,5-trisphosphate receptor interacting protein [Source:HGNC Symbol;Acc:HGNC:29370] | 1.107225354 | 1.324421195 |
| SPINT1 | serine peptidase inhibitor, Kunitz type 1 [Source:HGNC Symbol;Acc:HGNC:11246] | 1.110581105 | 2.354903902 |
| LRIG3 | leucine rich repeats and immunoglobulin like domains 3 [Source:HGNC Symbol;Acc:HGNC:30991] | 1.110806083 | 1.195127554 |
| FMNL1 | formin like 1 [Source:HGNC Symbol;Acc:HGNC:1212] | 1.12410864 | 1.347105504 |
| SBNO2 | strawberry notch homolog 2 [Source:HGNC Symbol;Acc:HGNC:29158] | 1.13151985 | 1.226232565 |
| BCL3 | B-cell CLL/lymphoma 3 [Source:HGNC Symbol;Acc:HGNC:998] | 1.148220995 | 1.148600056 |
| SH3BP1 | SH3 domain binding protein 1 [Source:HGNC Symbol;Acc:HGNC:10824] | 1.149466142 | 1.290107508 |
| GPR176 | G protein-coupled receptor 176 [Source:HGNC Symbol;Acc:HGNC:32370] | 1.157659475 | 1.511470143 |
| SLC26A11 | solute carrier family 26 member 11 [Source:HGNC Symbol;Acc:HGNC:14471] | 1.162732617 | 1.592610665 |
| AKR1B1 | aldo-keto reductase family 1 member B [Source:HGNC Symbol;Acc:HGNC:381] | 1.163487109 | 1.486843261 |
| STARD10 | StAR related lipid transfer domain containing 10 [Source:HGNC Symbol;Acc:HGNC:10666] | 1.169229532 | 1.97501027 |
| KSR1 | kinase suppressor of ras 1 [Source:HGNC Symbol;Acc:HGNC:6465] | 1.172495577 | 1.047563078 |
| LOC100862671 |  | 1.173003396 | 1.233193487 |
| PRKCD | protein kinase C delta [Source:HGNC Symbol;Acc:HGNC:9399] | 1.178342654 | 1.240471286 |
| GTPBP1 | GTP binding protein 1 [Source:HGNC Symbol;Acc:HGNC:4669] | 1.182050678 | 1.373326454 |
| TAPBP | TAP binding protein [Source:HGNC Symbol;Acc:HGNC:11566] | 1.193081186 | 1.121746598 |
| TNFRSF14 | TNF receptor superfamily member 14 [Source:HGNC Symbol;Acc:HGNC:11912] | 1.209328519 | 1.551479901 |
| AHRR | aryl-hydrocarbon receptor repressor [Source:HGNC Symbol;Acc:HGNC:346] | 1.210496432 | 2.781380562 |
| MXD1 | MAX dimerization protein 1 [Source:HGNC Symbol;Acc:HGNC:6761] | 1.220987887 | 1.27414117 |
| CEBPB | CCAAT/enhancer binding protein beta [Source:HGNC Symbol;Acc:HGNC:1834] | 1.224440484 | 1.971660401 |
| ALPK1 | alpha kinase 1 [Source:HGNC Symbol;Acc:HGNC:20917] | 1.229521611 | 1.401125965 |
| CELSR3 | cadherin EGF LAG seven-pass G-type receptor 3 [Source:HGNC Symbol;Acc:HGNC:3230] | 1.23486211 | 1.585344122 |
| BAMBI | BMP and activin membrane bound inhibitor [Source:HGNC Symbol;Acc:HGNC:30251] | 1.236395229 | 1.245963125 |
| ZNF267 | zinc finger protein 267 [Source:HGNC Symbol;Acc:HGNC:13060] | 1.238008043 | 1.119028912 |
| CEACAM19 | carcinoembryonic antigen related cell adhesion molecule 19 [Source:HGNC Symbol;Acc:HGNC:31951] | 1.241847628 | 1.275482272 |
| MMD | monocyte to macrophage differentiation associated [Source:HGNC Symbol;Acc:HGNC:7153] | 1.24386271 | 1.406731771 |
| NRP2 | neuropilin 2 [Source:HGNC Symbol;Acc:HGNC:8005] | 1.24794736 | 1.237536124 |
| HEXDC | hexosaminidase D [Source:HGNC Symbol;Acc:HGNC:26307] | 1.248689909 | 1.302034841 |
| C10orf10 | chromosome 10 open reading frame 10 [Source:HGNC Symbol;Acc:HGNC:23355] | 1.248865413 | 2.021192368 |
| SNX16 | sorting nexin 16 [Source:HGNC Symbol;Acc:HGNC:14980] | 1.249080159 | 1.288821825 |
| BRSK1 | BR serine/threonine kinase 1 [Source:HGNC Symbol;Acc:HGNC:18994] | 1.271752181 | 1.416469115 |
| TRIM25 | tripartite motif containing 25 [Source:HGNC Symbol;Acc:HGNC:12932] | 1.272686539 | 1.01814319 |
| TMEM178A | transmembrane protein 178A [Source:HGNC Symbol;Acc:HGNC:28517] | 1.278755292 | 2.119075331 |
| IRF2 | interferon regulatory factor 2 [Source:HGNC Symbol;Acc:HGNC:6117] | 1.278833874 | 1.266267931 |
| S1PR2 | sphingosine-1-phosphate receptor 2 [Source:HGNC Symbol;Acc:HGNC:3169] | 1.284873738 | 1.63308053 |
| DNAJB9 | DnaJ heat shock protein family (Hsp40) member B9 [Source:HGNC Symbol;Acc:HGNC:6968] | 1.294119487 | 1.328743426 |
| PCGF5 | polycomb group ring finger 5 [Source:HGNC Symbol;Acc:HGNC:28264] | 1.29902594 | 1.172877573 |
| EPS8L1 | EPS8 like 1 [Source:HGNC Symbol;Acc:HGNC:21295] | 1.299048701 | 1.472667393 |
| XRN1 | 5'-3' exoribonuclease 1 [Source:HGNC Symbol;Acc:HGNC:30654] | 1.30364596 | 1.190025588 |
| ETS2 | ETS proto-oncogene 2, transcription factor [Source:HGNC Symbol;Acc:HGNC:3489] | 1.308796759 | 1.498323314 |
| HLA-A | major histocompatibility complex, class I, A [Source:HGNC Symbol;Acc:HGNC:4931] | 1.311223481 | 1.795120686 |
| ADAR | adenosine deaminase, RNA specific [Source:HGNC Symbol;Acc:HGNC:225] | 1.320219803 | 1.328100947 |
| ETS1 | ETS proto-oncogene 1, transcription factor [Source:HGNC Symbol;Acc:HGNC:3488] | 1.322347011 | 1.240288461 |
| TMEM255A | transmembrane protein 255A [Source:HGNC Symbol;Acc:HGNC:26086] | 1.325204939 | 1.357463342 |
| NFIL3 | nuclear factor, interleukin 3 regulated [Source:HGNC Symbol;Acc:HGNC:7787] | 1.327326226 | 1.614500591 |
| HECA | hdc homolog, cell cycle regulator [Source:HGNC Symbol;Acc:HGNC:21041] | 1.350148577 | 1.939808981 |
| AGER | advanced glycosylation end-product specific receptor [Source:HGNC Symbol;Acc:HGNC:320] | 1.353425642 | 1.119288827 |
| SLC8B1 | solute carrier family 8 member B1 [Source:HGNC Symbol;Acc:HGNC:26175] | 1.356274506 | 1.272317406 |
| SLC2A13 | solute carrier family 2 member 13 [Source:HGNC Symbol;Acc:HGNC:15956] | 1.359149999 | 1.891777462 |
| MUC1 | mucin 1, cell surface associated [Source:HGNC Symbol;Acc:HGNC:7508] | 1.363767366 | 1.12993568 |
| LGALS3BP | galectin 3 binding protein [Source:HGNC Symbol;Acc:HGNC:6564] | 1.367465561 | 1.634909015 |
| MOB3C | MOB kinase activator 3C [Source:HGNC Symbol;Acc:HGNC:29800] | 1.368531621 | 1.282884123 |
| CA9 | carbonic anhydrase 9 [Source:HGNC Symbol;Acc:HGNC:1383] | 1.369008792 | 1.633250063 |
| CASP7 | caspase 7 [Source:HGNC Symbol;Acc:HGNC:1508] | 1.376346937 | 1.007916045 |
| HIST2H3D | histone cluster 2 H3 family member d [Source:HGNC Symbol;Acc:HGNC:25311] | 1.379585089 | 1.272079259 |
| STOM | stomatin [Source:HGNC Symbol;Acc:HGNC:3383] | 1.386019208 | 2.208609917 |
| WSB1 | WD repeat and SOCS box containing 1 [Source:HGNC Symbol;Acc:HGNC:19221] | 1.389372975 | 1.519234855 |
| CD27-AS1 | CD27 antisense RNA 1 [Source:HGNC Symbol;Acc:HGNC:43896] | 1.390927787 | 1.621519777 |
| DENND2D | DENN domain containing 2D [Source:HGNC Symbol;Acc:HGNC:26192] | 1.39133558 | 2.070013264 |
| STARD4 | StAR related lipid transfer domain containing 4 [Source:HGNC Symbol;Acc:HGNC:18058] | 1.392174135 | 1.009821265 |
| CARD11 | caspase recruitment domain family member 11 [Source:HGNC Symbol;Acc:HGNC:16393] | 1.393644108 | 1.460050029 |
| PGF | placental growth factor [Source:HGNC Symbol;Acc:HGNC:8893] | 1.408183822 | 1.518621345 |
| PER1 | period circadian clock 1 [Source:HGNC Symbol;Acc:HGNC:8845] | 1.408184487 | 2.06553176 |
| ERRFI1 | ERBB receptor feedback inhibitor 1 [Source:HGNC Symbol;Acc:HGNC:18185] | 1.411109766 | 1.67062303 |
| TNFAIP8 | TNF alpha induced protein 8 [Source:HGNC Symbol;Acc:HGNC:17260] | 1.411143207 | 2.065029037 |
| HIST2H2BA | histone cluster 2 H2B family member a (pseudogene) [Source:HGNC Symbol;Acc:HGNC:20560] | 1.421417668 | 1.494054162 |
| NUAK2 | NUAK family kinase 2 [Source:HGNC Symbol;Acc:HGNC:29558] | 1.430396114 | 1.269436135 |
| TMEM140 | transmembrane protein 140 [Source:HGNC Symbol;Acc:HGNC:21870] | 1.431070284 | 1.136548354 |
| GFPT2 | glutamine-fructose-6-phosphate transaminase 2 [Source:HGNC Symbol;Acc:HGNC:4242] | 1.433770855 | 1.655725386 |
| FBXO6 | F-box protein 6 [Source:HGNC Symbol;Acc:HGNC:13585] | 1.437892738 | 1.42632748 |
| GRIN2D | glutamate ionotropic receptor NMDA type subunit 2D [Source:HGNC Symbol;Acc:HGNC:4588] | 1.439994159 | 2.494487511 |
| CLDN4 | claudin 4 [Source:HGNC Symbol;Acc:HGNC:2046] | 1.446863476 | 1.966307713 |
| PRICKLE3 | prickle planar cell polarity protein 3 [Source:HGNC Symbol;Acc:HGNC:6645] | 1.448357359 | 1.668946313 |
| ZNF467 | zinc finger protein 467 [Source:HGNC Symbol;Acc:HGNC:23154] | 1.449864448 | 1.547540382 |
| RASSF4 | Ras association domain family member 4 [Source:HGNC Symbol;Acc:HGNC:20793] | 1.456197137 | 2.43686758 |
| HSPA2 | heat shock protein family A (Hsp70) member 2 [Source:HGNC Symbol;Acc:HGNC:5235] | 1.463298695 | 1.626053796 |
| SLC25A28 | solute carrier family 25 member 28 [Source:HGNC Symbol;Acc:HGNC:23472] | 1.464656246 | 1.782874607 |
| RHBDF2 | rhomboid 5 homolog 2 [Source:HGNC Symbol;Acc:HGNC:20788] | 1.474368104 | 1.201106437 |
| CITED4 | Cbp/p300 interacting transactivator with Glu/Asp rich carboxy-terminal domain 4 [Source:HGNC Symbol;Acc:HGNC:18696] | 1.474489012 | 1.358627544 |
| RNF19B | ring finger protein 19B [Source:HGNC Symbol;Acc:HGNC:26886] | 1.479894624 | 1.49023375 |
| PHF11 | PHD finger protein 11 [Source:HGNC Symbol;Acc:HGNC:17024] | 1.480115087 | 1.293376488 |
| CBX4 | chromobox 4 [Source:HGNC Symbol;Acc:HGNC:1554] | 1.488656442 | 1.428256324 |
| CHEK2 | checkpoint kinase 2 [Source:HGNC Symbol;Acc:HGNC:16627] | 1.500766842 | 1.508201865 |
| TNRC6C-AS1 | TNRC6C antisense RNA 1 [Source:HGNC Symbol;Acc:HGNC:44360] | 1.504182996 | 1.959170686 |
| ENO3 | enolase 3 [Source:HGNC Symbol;Acc:HGNC:3354] | 1.508368123 | 1.692173477 |
| ZNF296 | zinc finger protein 296 [Source:HGNC Symbol;Acc:HGNC:15981] | 1.509152204 | 1.518901487 |
| DRAM1 | DNA damage regulated autophagy modulator 1 [Source:HGNC Symbol;Acc:HGNC:25645] | 1.518419164 | 1.513620074 |
| ZC3H12C | zinc finger CCCH-type containing 12C [Source:HGNC Symbol;Acc:HGNC:29362] | 1.529579434 | 1.538856941 |
| LOC100506178 |  | 1.533969278 | 2.866542386 |
| RAD9A | RAD9 checkpoint clamp component A [Source:HGNC Symbol;Acc:HGNC:9827] | 1.544549216 | 1.708982426 |
| PML | promyelocytic leukemia [Source:HGNC Symbol;Acc:HGNC:9113] | 1.544684802 | 1.449004111 |
| PLEKHF1 | pleckstrin homology and FYVE domain containing 1 [Source:HGNC Symbol;Acc:HGNC:20764] | 1.545769955 | 1.860760941 |
| SLC39A8 | solute carrier family 39 member 8 [Source:HGNC Symbol;Acc:HGNC:20862] | 1.549582763 | 1.695617463 |
| NDRG1 | N-myc downstream regulated 1 [Source:HGNC Symbol;Acc:HGNC:7679] | 1.557396846 | 1.407599677 |
| ANKLE1 | ankyrin repeat and LEM domain containing 1 [Source:HGNC Symbol;Acc:HGNC:26812] | 1.564037858 | 1.65436272 |
| KDM7A | lysine demethylase 7A [Source:HGNC Symbol;Acc:HGNC:22224] | 1.568413181 | 2.206985909 |
| SLC43A3 | solute carrier family 43 member 3 [Source:HGNC Symbol;Acc:HGNC:17466] | 1.571274062 | 1.200847319 |
| GUCA1B | guanylate cyclase activator 1B [Source:HGNC Symbol;Acc:HGNC:4679] | 1.599907059 | 1.755838143 |
| CSF1 | colony stimulating factor 1 [Source:HGNC Symbol;Acc:HGNC:2432] | 1.608005389 | 1.353167529 |
| CAPN12 | calpain 12 [Source:HGNC Symbol;Acc:HGNC:13249] | 1.613039359 | 2.927811698 |
| HIP1R | huntingtin interacting protein 1 related [Source:HGNC Symbol;Acc:HGNC:18415] | 1.613928166 | 1.8076875 |
| PLEKHG6 | pleckstrin homology and RhoGEF domain containing G6 [Source:HGNC Symbol;Acc:HGNC:25562] | 1.635293285 | 2.84843203 |
| MFSD2A | major facilitator superfamily domain containing 2A [Source:HGNC Symbol;Acc:HGNC:25897] | 1.63685724 | 1.535002844 |
| SH2B3 | SH2B adaptor protein 3 [Source:HGNC Symbol;Acc:HGNC:29605] | 1.639505597 | 1.723689597 |
| MTMR11 | myotubularin related protein 11 [Source:HGNC Symbol;Acc:HGNC:24307] | 1.643511599 | 2.11755823 |
| PPP1R13L | protein phosphatase 1 regulatory subunit 13 like [Source:HGNC Symbol;Acc:HGNC:18838] | 1.657687294 | 2.042494716 |
| MMP25 | matrix metallopeptidase 25 [Source:HGNC Symbol;Acc:HGNC:14246] | 1.661838611 | 2.302843904 |
| ERO1LB |  | 1.667181078 | 1.981114389 |
| IL18BP | interleukin 18 binding protein [Source:HGNC Symbol;Acc:HGNC:5987] | 1.672417945 | 1.61463768 |
| C17orf96 | chromosome 17 open reading frame 96 [Source:HGNC Symbol;Acc:HGNC:34493] | 1.678618776 | 1.374092446 |
| HLA-E | major histocompatibility complex, class I, E [Source:HGNC Symbol;Acc:HGNC:4962] | 1.682537793 | 1.801000664 |
| OPTN | optineurin [Source:HGNC Symbol;Acc:HGNC:17142] | 1.698412331 | 1.21757885 |
| EML2 | echinoderm microtubule associated protein like 2 [Source:HGNC Symbol;Acc:HGNC:18035] | 1.717128935 | 2.451546149 |
| PTX3 | pentraxin 3 [Source:HGNC Symbol;Acc:HGNC:9692] | 1.721218793 | 1.726490444 |
| TXNIP | thioredoxin interacting protein [Source:HGNC Symbol;Acc:HGNC:16952] | 1.722765488 | 3.404029801 |
| MICB | MHC class I polypeptide-related sequence B [Source:HGNC Symbol;Acc:HGNC:7091] | 1.723007584 | 1.978640786 |
| OGFR-AS1 | OGFR antisense RNA 1 [Source:HGNC Symbol;Acc:HGNC:40724] | 1.72381702 | 2.072933119 |
| MISP | mitotic spindle positioning [Source:HGNC Symbol;Acc:HGNC:27000] | 1.75423632 | 2.264139524 |
| CREBRF | CREB3 regulatory factor [Source:HGNC Symbol;Acc:HGNC:24050] | 1.785674276 | 2.090477102 |
| ZFP36 | ZFP36 ring finger protein [Source:HGNC Symbol;Acc:HGNC:12862] | 1.785857865 | 1.371253378 |
| PNPT1 | polyribonucleotide nucleotidyltransferase 1 [Source:HGNC Symbol;Acc:HGNC:23166] | 1.788333402 | 1.214542185 |
| HES4 | hes family bHLH transcription factor 4 [Source:HGNC Symbol;Acc:HGNC:24149] | 1.789602173 | 2.09929795 |
| VEGFC | vascular endothelial growth factor C [Source:HGNC Symbol;Acc:HGNC:12682] | 1.799217264 | 2.371229276 |
| TRANK1 | tetratricopeptide repeat and ankyrin repeat containing 1 [Source:HGNC Symbol;Acc:HGNC:29011] | 1.799237214 | 2.0012076 |
| BRICD5 | BRICHOS domain containing 5 [Source:HGNC Symbol;Acc:HGNC:28309] | 1.808275713 | 2.478751398 |
| TNIP1 | TNFAIP3 interacting protein 1 [Source:HGNC Symbol;Acc:HGNC:16903] | 1.832182538 | 1.510539064 |
| NAGS | N-acetylglutamate synthase [Source:HGNC Symbol;Acc:HGNC:17996] | 1.847149303 | 2.615157436 |
| STAT5A | signal transducer and activator of transcription 5A [Source:HGNC Symbol;Acc:HGNC:11366] | 1.859675054 | 2.55529917 |
| PPP4R1L | protein phosphatase 4 regulatory subunit 1 like (pseudogene) [Source:HGNC Symbol;Acc:HGNC:15755] | 1.867549156 | 1.963952821 |
| KCNQ4 | potassium voltage-gated channel subfamily Q member 4 [Source:HGNC Symbol;Acc:HGNC:6298] | 1.868038523 | 2.503780525 |
| PDE4B | phosphodiesterase 4B [Source:HGNC Symbol;Acc:HGNC:8781] | 1.873943662 | 2.034918936 |
| ADAMTS4 | ADAM metallopeptidase with thrombospondin type 1 motif 4 [Source:HGNC Symbol;Acc:HGNC:220] | 1.876477836 | 1.835506331 |
| GMPR | guanosine monophosphate reductase [Source:HGNC Symbol;Acc:HGNC:4376] | 1.878974097 | 1.377867467 |
| ANGPT2 | angiopoietin 2 [Source:HGNC Symbol;Acc:HGNC:485] | 1.885176227 | 1.858183338 |
| LRRC75A | leucine rich repeat containing 75A [Source:HGNC Symbol;Acc:HGNC:32403] | 1.886518135 | 1.983927191 |
| IFNLR1 | interferon lambda receptor 1 [Source:HGNC Symbol;Acc:HGNC:18584] | 1.89457664 | 1.918971159 |
| NYAP1 | neuronal tyrosine phosphorylated phosphoinositide-3-kinase adaptor 1 [Source:HGNC Symbol;Acc:HGNC:22009] | 1.914490362 | 2.587236051 |
| EFNA1 | ephrin A1 [Source:HGNC Symbol;Acc:HGNC:3221] | 1.91540746 | 2.523127546 |
| STAT2 | signal transducer and activator of transcription 2 [Source:HGNC Symbol;Acc:HGNC:11363] | 1.915459445 | 1.753352272 |
| C8orf46 | chromosome 8 open reading frame 46 [Source:HGNC Symbol;Acc:HGNC:28498] | 1.915714747 | 1.346739748 |
| ANKRD33B | ankyrin repeat domain 33B [Source:HGNC Symbol;Acc:HGNC:35240] | 1.932130388 | 1.728517858 |
| TMEM229B | transmembrane protein 229B [Source:HGNC Symbol;Acc:HGNC:20130] | 1.940143456 | 2.039827709 |
| BPGM | bisphosphoglycerate mutase [Source:HGNC Symbol;Acc:HGNC:1093] | 1.946110438 | 2.058788387 |
| TRABD2A | TraB domain containing 2A [Source:HGNC Symbol;Acc:HGNC:27013] | 1.951462377 | 1.914114065 |
| ARID5A | AT-rich interaction domain 5A [Source:HGNC Symbol;Acc:HGNC:17361] | 1.957150791 | 1.336453862 |
| APOL2 | apolipoprotein L2 [Source:HGNC Symbol;Acc:HGNC:619] | 1.958213109 | 1.269141736 |
| CCL2 | C-C motif chemokine ligand 2 [Source:HGNC Symbol;Acc:HGNC:10618] | 1.977005367 | 3.079156034 |
| CCDC114 | coiled-coil domain containing 114 [Source:HGNC Symbol;Acc:HGNC:26560] | 1.980566641 | 2.503125992 |
| SQRDL | sulfide quinone reductase-like (yeast) [Source:HGNC Symbol;Acc:HGNC:20390] | 1.994587778 | 2.483228869 |
| RORB | RAR related orphan receptor B [Source:HGNC Symbol;Acc:HGNC:10259] | 1.995112119 | 1.498210208 |
| MAP2 | microtubule associated protein 2 [Source:HGNC Symbol;Acc:HGNC:6839] | 2.004957048 | 2.7317562 |
| HAPLN3 | hyaluronan and proteoglycan link protein 3 [Source:HGNC Symbol;Acc:HGNC:21446] | 2.012035177 | 2.267549641 |
| APOBEC3F | apolipoprotein B mRNA editing enzyme catalytic subunit 3F [Source:HGNC Symbol;Acc:HGNC:17356] | 2.030570228 | 1.720109821 |
| TIFA | TRAF interacting protein with forkhead associated domain [Source:HGNC Symbol;Acc:HGNC:19075] | 2.032110194 | 2.12939637 |
| SP100 | SP100 nuclear antigen [Source:HGNC Symbol;Acc:HGNC:11206] | 2.03621208 | 1.897100574 |
| RNF213 | ring finger protein 213 [Source:HGNC Symbol;Acc:HGNC:14539] | 2.037553226 | 1.831965202 |
| SYNGR3 | synaptogyrin 3 [Source:HGNC Symbol;Acc:HGNC:11501] | 2.03756353 | 3.079679247 |
| IFITM2 | interferon induced transmembrane protein 2 [Source:HGNC Symbol;Acc:HGNC:5413] | 2.040633905 | 1.34740071 |
| OVGP1 | oviductal glycoprotein 1 [Source:HGNC Symbol;Acc:HGNC:8524] | 2.04285409 | 2.392246429 |
| ZC3HAV1 | zinc finger CCCH-type containing, antiviral 1 [Source:HGNC Symbol;Acc:HGNC:23721] | 2.060301148 | 1.979054072 |
| TMEM62 | transmembrane protein 62 [Source:HGNC Symbol;Acc:HGNC:26269] | 2.070544593 | 1.346961077 |
| CEBPD | CCAAT/enhancer binding protein delta [Source:HGNC Symbol;Acc:HGNC:1835] | 2.085694611 | 2.543569304 |
| HLA-J | major histocompatibility complex, class I, J (pseudogene) [Source:HGNC Symbol;Acc:HGNC:4967] | 2.086193825 | 2.272975432 |
| JUNB | JunB proto-oncogene, AP-1 transcription factor subunit [Source:HGNC Symbol;Acc:HGNC:6205] | 2.090582899 | 1.745032853 |
| LOC101927204 |  | 2.097904587 | 2.117217588 |
| CXCL16 | C-X-C motif chemokine ligand 16 [Source:HGNC Symbol;Acc:HGNC:16642] | 2.113838741 | 1.441554713 |
| KLHL15 | kelch like family member 15 [Source:HGNC Symbol;Acc:HGNC:29347] | 2.129494007 | 1.420472188 |
| MR1 | major histocompatibility complex, class I-related [Source:HGNC Symbol;Acc:HGNC:4975] | 2.148206601 | 2.116666165 |
| COL16A1 | collagen type XVI alpha 1 chain [Source:HGNC Symbol;Acc:HGNC:2193] | 2.150435428 | 2.652431232 |
| EIF2AK2 | eukaryotic translation initiation factor 2 alpha kinase 2 [Source:HGNC Symbol;Acc:HGNC:9437] | 2.165709638 | 2.439927369 |
| PELI1 | pellino E3 ubiquitin protein ligase 1 [Source:HGNC Symbol;Acc:HGNC:8827] | 2.175289372 | 2.40927501 |
| NFKB2 | nuclear factor kappa B subunit 2 [Source:HGNC Symbol;Acc:HGNC:7795] | 2.179102265 | 1.99374719 |
| MAFF | MAF bZIP transcription factor F [Source:HGNC Symbol;Acc:HGNC:6780] | 2.180738525 | 2.136815212 |
| IL15 | interleukin 15 [Source:HGNC Symbol;Acc:HGNC:5977] | 2.183822126 | 1.968256205 |
| PSMB8 | proteasome subunit beta 8 [Source:HGNC Symbol;Acc:HGNC:9545] | 2.188231307 | 1.619012115 |
| OTUD1 | OTU deubiquitinase 1 [Source:HGNC Symbol;Acc:HGNC:27346] | 2.212265304 | 2.562682991 |
| SERPING1 | serpin family G member 1 [Source:HGNC Symbol;Acc:HGNC:1228] | 2.222967047 | 2.447385534 |
| PIK3AP1 | phosphoinositide-3-kinase adaptor protein 1 [Source:HGNC Symbol;Acc:HGNC:30034] | 2.227721926 | 1.848528292 |
| TAPBPL | TAP binding protein like [Source:HGNC Symbol;Acc:HGNC:30683] | 2.230538228 | 1.528040646 |
| C7orf61 | chromosome 7 open reading frame 61 [Source:HGNC Symbol;Acc:HGNC:22135] | 2.236726701 | 2.923248808 |
| ATG16L2 | autophagy related 16 like 2 [Source:HGNC Symbol;Acc:HGNC:25464] | 2.238927631 | 3.047992906 |
| PPP1R15A | protein phosphatase 1 regulatory subunit 15A [Source:HGNC Symbol;Acc:HGNC:14375] | 2.251907051 | 1.472327698 |
| IFITM3 | interferon induced transmembrane protein 3 [Source:HGNC Symbol;Acc:HGNC:5414] | 2.268126257 | 1.984586911 |
| DDIT3 | DNA damage inducible transcript 3 [Source:HGNC Symbol;Acc:HGNC:2726] | 2.278064919 | 1.997610964 |
| CNTNAP1 | contactin associated protein 1 [Source:HGNC Symbol;Acc:HGNC:8011] | 2.281215166 | 2.567524559 |
| PDZD2 | PDZ domain containing 2 [Source:HGNC Symbol;Acc:HGNC:18486] | 2.285725596 | 1.529279053 |
| IL7R | interleukin 7 receptor [Source:HGNC Symbol;Acc:HGNC:6024] | 2.288412305 | 1.856006009 |
| ATP10A | ATPase phospholipid transporting 10A (putative) [Source:HGNC Symbol;Acc:HGNC:13542] | 2.320619152 | 1.359295294 |
| SLC1A3 | solute carrier family 1 member 3 [Source:HGNC Symbol;Acc:HGNC:10941] | 2.323568842 | 2.113014859 |
| ACKR3 | atypical chemokine receptor 3 [Source:HGNC Symbol;Acc:HGNC:23692] | 2.335721344 | 2.157266626 |
| CYP2J2 | cytochrome P450 family 2 subfamily J member 2 [Source:HGNC Symbol;Acc:HGNC:2634] | 2.342510475 | 1.567136377 |
| PLEK2 | pleckstrin 2 [Source:HGNC Symbol;Acc:HGNC:19238] | 2.34455327 | 2.438685033 |
| TMEM92 | transmembrane protein 92 [Source:HGNC Symbol;Acc:HGNC:26579] | 2.346369774 | 2.285922952 |
| CDC42EP5 | CDC42 effector protein 5 [Source:HGNC Symbol;Acc:HGNC:17408] | 2.359387926 | 2.834865197 |
| ERN1 | endoplasmic reticulum to nucleus signaling 1 [Source:HGNC Symbol;Acc:HGNC:3449] | 2.359401102 | 2.011308392 |
| GCH1 | GTP cyclohydrolase 1 [Source:HGNC Symbol;Acc:HGNC:4193] | 2.371337942 | 1.986505962 |
| HLA-C | major histocompatibility complex, class I, C [Source:HGNC Symbol;Acc:HGNC:4933] | 2.424258188 | 2.614579419 |
| SIPA1L2 | signal induced proliferation associated 1 like 2 [Source:HGNC Symbol;Acc:HGNC:23800] | 2.444871063 | 3.098033417 |
| IFIT5 | interferon induced protein with tetratricopeptide repeats 5 [Source:HGNC Symbol;Acc:HGNC:13328] | 2.446276099 | 1.965160006 |
| PPM1K | protein phosphatase, Mg2+/Mn2+ dependent 1K [Source:HGNC Symbol;Acc:HGNC:25415] | 2.464703976 | 2.613192436 |
| PMAIP1 | phorbol-12-myristate-13-acetate-induced protein 1 [Source:HGNC Symbol;Acc:HGNC:9108] | 2.487007986 | 2.163965737 |
| B3GNT7 | UDP-GlcNAc:betaGal beta-1,3-N-acetylglucosaminyltransferase 7 [Source:HGNC Symbol;Acc:HGNC:18811] | 2.489370211 | 3.364121826 |
| H1F0 | H1 histone family member 0 [Source:HGNC Symbol;Acc:HGNC:4714] | 2.495251817 | 3.449480408 |
| PKP1 | plakophilin 1 [Source:HGNC Symbol;Acc:HGNC:9023] | 2.515967264 | 2.033194247 |
| HLA-DOB | major histocompatibility complex, class II, DO beta [Source:HGNC Symbol;Acc:HGNC:4937] | 2.516817968 | 1.975726565 |
| TAP2 | transporter 2, ATP binding cassette subfamily B member [Source:HGNC Symbol;Acc:HGNC:44] | 2.547249544 | 1.447167962 |
| WDR65 |  | 2.558515123 | 2.785696297 |
| LY6E | lymphocyte antigen 6 complex, locus E [Source:HGNC Symbol;Acc:HGNC:6727] | 2.568986231 | 2.540703037 |
| PTGS2 | prostaglandin-endoperoxide synthase 2 [Source:HGNC Symbol;Acc:HGNC:9605] | 2.585017899 | 3.083096441 |
| NLRC5 | NLR family CARD domain containing 5 [Source:HGNC Symbol;Acc:HGNC:29933] | 2.605311903 | 1.899735718 |
| ARHGEF16 | Rho guanine nucleotide exchange factor 16 [Source:HGNC Symbol;Acc:HGNC:15515] | 2.626607022 | 3.28065883 |
| NFKBIE | NFKB inhibitor epsilon [Source:HGNC Symbol;Acc:HGNC:7799] | 2.631817578 | 2.53185989 |
| TRIM14 | tripartite motif containing 14 [Source:HGNC Symbol;Acc:HGNC:16283] | 2.665461639 | 1.999069272 |
| N4BP3 | NEDD4 binding protein 3 [Source:HGNC Symbol;Acc:HGNC:29852] | 2.680290094 | 3.021512525 |
| CGB7 | chorionic gonadotropin beta subunit 7 [Source:HGNC Symbol;Acc:HGNC:16451] | 2.687626682 | 2.874371178 |
| IL15RA | interleukin 15 receptor subunit alpha [Source:HGNC Symbol;Acc:HGNC:5978] | 2.703897066 | 2.565935772 |
| GBP2 | guanylate binding protein 2 [Source:HGNC Symbol;Acc:HGNC:4183] | 2.705446858 | 2.474951386 |
| C19orf66 | chromosome 19 open reading frame 66 [Source:HGNC Symbol;Acc:HGNC:25649] | 2.711490388 | 1.999672336 |
| B2M | beta-2-microglobulin [Source:HGNC Symbol;Acc:HGNC:914] | 2.71583754 | 3.360510128 |
| NFKBIA | NFKB inhibitor alpha [Source:HGNC Symbol;Acc:HGNC:7797] | 2.722981204 | 3.493702749 |
| FST | follistatin [Source:HGNC Symbol;Acc:HGNC:3971] | 2.742079554 | 2.559426036 |
| IRAK2 | interleukin 1 receptor associated kinase 2 [Source:HGNC Symbol;Acc:HGNC:6113] | 2.743140167 | 3.181639994 |
| TDRD7 | tudor domain containing 7 [Source:HGNC Symbol;Acc:HGNC:30831] | 2.75544989 | 2.459515006 |
| CES3 | carboxylesterase 3 [Source:HGNC Symbol;Acc:HGNC:1865] | 2.812559432 | 2.933040182 |
| APLF | aprataxin and PNKP like factor [Source:HGNC Symbol;Acc:HGNC:28724] | 2.835967095 | 2.173866649 |
| C1S | complement C1s [Source:HGNC Symbol;Acc:HGNC:1247] | 2.84654942 | 2.777163931 |
| CCR10 | C-C motif chemokine receptor 10 [Source:HGNC Symbol;Acc:HGNC:4474] | 2.912852338 | 3.638715693 |
| C1QTNF1 | C1q and tumor necrosis factor related protein 1 [Source:HGNC Symbol;Acc:HGNC:14324] | 2.919580812 | 3.158917937 |
| TRIM21 | tripartite motif containing 21 [Source:HGNC Symbol;Acc:HGNC:11312] | 2.982374279 | 2.020351507 |
| ZC3H12A | zinc finger CCCH-type containing 12A [Source:HGNC Symbol;Acc:HGNC:26259] | 2.990960603 | 3.366616571 |
| DTX3L | deltex E3 ubiquitin ligase 3L [Source:HGNC Symbol;Acc:HGNC:30323] | 3.01085056 | 2.845822297 |
| ACTN2 | actinin alpha 2 [Source:HGNC Symbol;Acc:HGNC:164] | 3.027205563 | 2.522282931 |
| HCP5 | HLA complex P5 (non-protein coding) [Source:HGNC Symbol;Acc:HGNC:21659] | 3.027855155 | 3.404472445 |
| CPEB3 | cytoplasmic polyadenylation element binding protein 3 [Source:HGNC Symbol;Acc:HGNC:21746] | 3.030138052 | 2.041377357 |
| PLA2G4C | phospholipase A2 group IVC [Source:HGNC Symbol;Acc:HGNC:9037] | 3.058735479 | 3.779724911 |
| KRT17 | keratin 17 [Source:HGNC Symbol;Acc:HGNC:6427] | 3.105979545 | 3.551334777 |
| CD40 | CD40 molecule [Source:HGNC Symbol;Acc:HGNC:11919] | 3.107815866 | 3.185618913 |
| BTN3A3 | butyrophilin subfamily 3 member A3 [Source:HGNC Symbol;Acc:HGNC:1140] | 3.109593035 | 3.144183199 |
| RARRES3 | retinoic acid receptor responder 3 [Source:HGNC Symbol;Acc:HGNC:9869] | 3.134154199 | 1.877076107 |
| BTN3A2 | butyrophilin subfamily 3 member A2 [Source:HGNC Symbol;Acc:HGNC:1139] | 3.152960383 | 3.423396596 |
| ABCG1 | ATP binding cassette subfamily G member 1 [Source:HGNC Symbol;Acc:HGNC:73] | 3.155899318 | 2.700841695 |
| KIAA1045 |  | 3.163775548 | 3.116683205 |
| SCO2 | SCO2, cytochrome c oxidase assembly protein [Source:HGNC Symbol;Acc:HGNC:10604] | 3.192563069 | 3.147487978 |
| HCG26 |  | 3.233131304 | 3.923684054 |
| BTN3A1 | butyrophilin subfamily 3 member A1 [Source:HGNC Symbol;Acc:HGNC:1138] | 3.315631687 | 3.311275936 |
| MAPK8IP2 | mitogen-activated protein kinase 8 interacting protein 2 [Source:HGNC Symbol;Acc:HGNC:6883] | 3.323642527 | 2.558975567 |
| NMI | N-myc and STAT interactor [Source:HGNC Symbol;Acc:HGNC:7854] | 3.341588298 | 2.247000565 |
| RHEBL1 | Ras homolog enriched in brain like 1 [Source:HGNC Symbol;Acc:HGNC:21166] | 3.349569942 | 3.363934885 |
| IRF1 | interferon regulatory factor 1 [Source:HGNC Symbol;Acc:HGNC:6116] | 3.360874827 | 2.697574011 |
| SOD2 | superoxide dismutase 2, mitochondrial [Source:HGNC Symbol;Acc:HGNC:11180] | 3.374111284 | 4.504815434 |
| HES7 | hes family bHLH transcription factor 7 [Source:HGNC Symbol;Acc:HGNC:15977] | 3.379625028 | 5.520000111 |
| MAP3K8 | mitogen-activated protein kinase kinase kinase 8 [Source:HGNC Symbol;Acc:HGNC:6860] | 3.409075948 | 3.505719353 |
| PLEKHA4 | pleckstrin homology domain containing A4 [Source:HGNC Symbol;Acc:HGNC:14339] | 3.411652388 | 2.537523538 |
| HLA-H | major histocompatibility complex, class I, H (pseudogene) [Source:HGNC Symbol;Acc:HGNC:4965] | 3.416355221 | 2.917329394 |
| SAMHD1 | SAM and HD domain containing deoxynucleoside triphosphate triphosphohydrolase 1 [Source:HGNC Symbol;Acc:HGNC:15925] | 3.451482485 | 2.739821576 |
| REM2 | RRAD and GEM like GTPase 2 [Source:HGNC Symbol;Acc:HGNC:20248] | 3.456630271 | 3.02810874 |
| PARP12 | poly(ADP-ribose) polymerase family member 12 [Source:HGNC Symbol;Acc:HGNC:21919] | 3.462120081 | 3.102177229 |
| IFI16 | interferon gamma inducible protein 16 [Source:HGNC Symbol;Acc:HGNC:5395] | 3.485163227 | 2.838087657 |
| STAT1 | signal transducer and activator of transcription 1 [Source:HGNC Symbol;Acc:HGNC:11362] | 3.513016114 | 3.042052132 |
| ADM2 | adrenomedullin 2 [Source:HGNC Symbol;Acc:HGNC:28898] | 3.669735272 | 3.813374375 |
| C2CD4A | C2 calcium dependent domain containing 4A [Source:HGNC Symbol;Acc:HGNC:33627] | 3.709183426 | 2.825379649 |
| KCNN1 | potassium calcium-activated channel subfamily N member 1 [Source:HGNC Symbol;Acc:HGNC:6290] | 3.737576274 | 3.80087467 |
| TAP1 | transporter 1, ATP binding cassette subfamily B member [Source:HGNC Symbol;Acc:HGNC:43] | 3.747223014 | 2.860205718 |
| FLT3LG | fms related tyrosine kinase 3 ligand [Source:HGNC Symbol;Acc:HGNC:3766] | 3.776150152 | 4.064658445 |
| ZSCAN4 | zinc finger and SCAN domain containing 4 [Source:HGNC Symbol;Acc:HGNC:23709] | 3.788109767 | 4.096614053 |
| RELB | RELB proto-oncogene, NF-kB subunit [Source:HGNC Symbol;Acc:HGNC:9956] | 3.804862853 | 3.587000771 |
| ACHE | acetylcholinesterase (Cartwright blood group) [Source:HGNC Symbol;Acc:HGNC:108] | 3.811244827 | 3.271309707 |
| CP | ceruloplasmin [Source:HGNC Symbol;Acc:HGNC:2295] | 3.813624409 | 3.360028601 |
| PLSCR1 | phospholipid scramblase 1 [Source:HGNC Symbol;Acc:HGNC:9092] | 3.856810094 | 3.635257252 |
| C1R | complement C1r [Source:HGNC Symbol;Acc:HGNC:1246] | 3.857621628 | 4.130149946 |
| TRIM69 | tripartite motif containing 69 [Source:HGNC Symbol;Acc:HGNC:17857] | 3.88696818 | 3.603600004 |
| ANO9 | anoctamin 9 [Source:HGNC Symbol;Acc:HGNC:20679] | 3.910748222 | 4.65236911 |
| HEY2 | hes related family bHLH transcription factor with YRPW motif 2 [Source:HGNC Symbol;Acc:HGNC:4881] | 3.917058606 | 3.159264049 |
| IRF7 | interferon regulatory factor 7 [Source:HGNC Symbol;Acc:HGNC:6122] | 3.923751248 | 3.486289639 |
| TNFSF13B | tumor necrosis factor superfamily member 13b [Source:HGNC Symbol;Acc:HGNC:11929] | 3.932320362 | 2.81283982 |
| PARP9 | poly(ADP-ribose) polymerase family member 9 [Source:HGNC Symbol;Acc:HGNC:24118] | 3.950601755 | 3.698584145 |
| RRAD | RRAD, Ras related glycolysis inhibitor and calcium channel regulator [Source:HGNC Symbol;Acc:HGNC:10446] | 3.953357084 | 3.954379088 |
| TNFSF9 | tumor necrosis factor superfamily member 9 [Source:HGNC Symbol;Acc:HGNC:11939] | 4.006927041 | 3.932652299 |
| SP8 | Sp8 transcription factor [Source:HGNC Symbol;Acc:HGNC:19196] | 4.018174819 | 4.154104608 |
| PATL2 | PAT1 homolog 2 [Source:HGNC Symbol;Acc:HGNC:33630] | 4.040095217 | 3.862528047 |
| KLF4 | Kruppel like factor 4 [Source:HGNC Symbol;Acc:HGNC:6348] | 4.114280683 | 3.898598026 |
| LGI4 | leucine rich repeat LGI family member 4 [Source:HGNC Symbol;Acc:HGNC:18712] | 4.19416105 | 5.22923667 |
| HELZ2 | helicase with zinc finger 2 [Source:HGNC Symbol;Acc:HGNC:30021] | 4.195982833 | 3.900479971 |
| ELF3 | E74 like ETS transcription factor 3 [Source:HGNC Symbol;Acc:HGNC:3318] | 4.238340972 | 4.149046153 |
| GRIP2 | glutamate receptor interacting protein 2 [Source:HGNC Symbol;Acc:HGNC:23841] | 4.260072096 | 3.733045944 |
| APOBEC3G | apolipoprotein B mRNA editing enzyme catalytic subunit 3G [Source:HGNC Symbol;Acc:HGNC:17357] | 4.310694849 | 3.61728735 |
| FOXA3 | forkhead box A3 [Source:HGNC Symbol;Acc:HGNC:5023] | 4.328771024 | 3.836948603 |
| PARP14 | poly(ADP-ribose) polymerase family member 14 [Source:HGNC Symbol;Acc:HGNC:29232] | 4.376573626 | 4.008348774 |
| THEMIS2 | thymocyte selection associated family member 2 [Source:HGNC Symbol;Acc:HGNC:16839] | 4.50391662 | 4.710009326 |
| HLA-B | major histocompatibility complex, class I, B [Source:HGNC Symbol;Acc:HGNC:4932] | 4.523701872 | 5.111968566 |
| IFI35 | interferon induced protein 35 [Source:HGNC Symbol;Acc:HGNC:5399] | 4.537713411 | 2.737583249 |
| ICAM5 | intercellular adhesion molecule 5 [Source:HGNC Symbol;Acc:HGNC:5348] | 4.540750197 | 6.275166355 |
| DDX60L | DEAD-box helicase 60-like [Source:HGNC Symbol;Acc:HGNC:26429] | 4.546587424 | 3.362493442 |
| TNFRSF9 | TNF receptor superfamily member 9 [Source:HGNC Symbol;Acc:HGNC:11924] | 4.567793152 | 2.571280183 |
| ICAM1 | intercellular adhesion molecule 1 [Source:HGNC Symbol;Acc:HGNC:5344] | 4.585419726 | 4.425540225 |
| TLR2 | toll like receptor 2 [Source:HGNC Symbol;Acc:HGNC:11848] | 4.641909531 | 4.56134686 |
| PARP10 | poly(ADP-ribose) polymerase family member 10 [Source:HGNC Symbol;Acc:HGNC:25895] | 4.671578612 | 4.046663258 |
| IFI44L | interferon induced protein 44 like [Source:HGNC Symbol;Acc:HGNC:17817] | 4.692055236 | 4.844324737 |
| P2RX7 | purinergic receptor P2X 7 [Source:HGNC Symbol;Acc:HGNC:8537] | 4.711577473 | 4.007285458 |
| TNFAIP2 | TNF alpha induced protein 2 [Source:HGNC Symbol;Acc:HGNC:11895] | 4.748154276 | 4.406334125 |
| LOC100505622 |  | 4.749891309 | 5.60886749 |
| CX3CL1 | C-X3-C motif chemokine ligand 1 [Source:HGNC Symbol;Acc:HGNC:10647] | 4.772319621 | 4.849712317 |
| CFB | complement factor B [Source:HGNC Symbol;Acc:HGNC:1037] | 4.783905695 | 4.955807912 |
| ZNF385C | zinc finger protein 385C [Source:HGNC Symbol;Acc:HGNC:33722] | 4.802723228 | 5.072273151 |
| HSH2D | hematopoietic SH2 domain containing [Source:HGNC Symbol;Acc:HGNC:24920] | 4.879223488 | 4.377267379 |
| DHX58 | DExH-box helicase 58 [Source:HGNC Symbol;Acc:HGNC:29517] | 4.88189842 | 4.394989493 |
| APOL6 | apolipoprotein L6 [Source:HGNC Symbol;Acc:HGNC:14870] | 4.901429748 | 3.653022895 |
| UBA7 | ubiquitin like modifier activating enzyme 7 [Source:HGNC Symbol;Acc:HGNC:12471] | 4.921199559 | 4.468406128 |
| MATN1 | matrilin 1, cartilage matrix protein [Source:HGNC Symbol;Acc:HGNC:6907] | 4.935724599 | 3.439365213 |
| TNFAIP3 | TNF alpha induced protein 3 [Source:HGNC Symbol;Acc:HGNC:11896] | 5.092662452 | 5.511841998 |
| NPTX1 | neuronal pentraxin 1 [Source:HGNC Symbol;Acc:HGNC:7952] | 5.124500441 | 4.683144083 |
| VCAM1 | vascular cell adhesion molecule 1 [Source:HGNC Symbol;Acc:HGNC:12663] | 5.14714039 | 5.375083661 |
| IL32 | interleukin 32 [Source:HGNC Symbol;Acc:HGNC:16830] | 5.202983269 | 5.369514876 |
| HEATR9 | HEAT repeat containing 9 [Source:HGNC Symbol;Acc:HGNC:26548] | 5.258390138 | 4.910287835 |
| IDO1 | indoleamine 2,3-dioxygenase 1 [Source:HGNC Symbol;Acc:HGNC:6059] | 5.265074214 | 3.501515335 |
| BIRC3 | baculoviral IAP repeat containing 3 [Source:HGNC Symbol;Acc:HGNC:591] | 5.296177618 | 5.793822644 |
| DDX58 | DExD/H-box helicase 58 [Source:HGNC Symbol;Acc:HGNC:19102] | 5.336196044 | 4.253488023 |
| UBE2L6 | ubiquitin conjugating enzyme E2 L6 [Source:HGNC Symbol;Acc:HGNC:12490] | 5.337576794 | 4.737833411 |
| HERC6 | HECT and RLD domain containing E3 ubiquitin protein ligase family member 6 [Source:HGNC Symbol;Acc:HGNC:26072] | 5.370252313 | 4.536742419 |
| SECTM1 | secreted and transmembrane 1 [Source:HGNC Symbol;Acc:HGNC:10707] | 5.538733478 | 4.583928137 |
| TNFAIP6 | TNF alpha induced protein 6 [Source:HGNC Symbol;Acc:HGNC:11898] | 5.553961014 | 7.109061576 |
| BATF3 | basic leucine zipper ATF-like transcription factor 3 [Source:HGNC Symbol;Acc:HGNC:28915] | 5.58535595 | 4.520028124 |
| CXCL2 | C-X-C motif chemokine ligand 2 [Source:HGNC Symbol;Acc:HGNC:4603] | 5.658496471 | 5.765809809 |
| LTB | lymphotoxin beta [Source:HGNC Symbol;Acc:HGNC:6711] | 5.719465149 | 6.216703544 |
| SCN3A | sodium voltage-gated channel alpha subunit 3 [Source:HGNC Symbol;Acc:HGNC:10590] | 5.721512399 | 5.330264485 |
| CXCL3 | C-X-C motif chemokine ligand 3 [Source:HGNC Symbol;Acc:HGNC:4604] | 5.742495204 | 5.077318705 |
| C3 | complement C3 [Source:HGNC Symbol;Acc:HGNC:1318] | 5.763128215 | 5.926703814 |
| C15orf48 | chromosome 15 open reading frame 48 [Source:HGNC Symbol;Acc:HGNC:29898] | 5.811817129 | 4.98158934 |
| ISG20 | interferon stimulated exonuclease gene 20 [Source:HGNC Symbol;Acc:HGNC:6130] | 5.901059139 | 4.197533514 |
| CXCL6 | C-X-C motif chemokine ligand 6 [Source:HGNC Symbol;Acc:HGNC:10643] | 5.907754116 | 6.093444399 |
| KCNV1 | potassium voltage-gated channel modifier subfamily V member 1 [Source:HGNC Symbol;Acc:HGNC:18861] | 5.915909235 | 5.482077547 |
| IL6 | interleukin 6 [Source:HGNC Symbol;Acc:HGNC:6018] | 5.980805199 | 7.41641948 |
| IL4I1 | interleukin 4 induced 1 [Source:HGNC Symbol;Acc:HGNC:19094] | 5.994121179 | 5.12911802 |
| C11orf96 | chromosome 11 open reading frame 96 [Source:HGNC Symbol;Acc:HGNC:38675] | 5.996359252 | 5.504143784 |
| IFIT3 | interferon induced protein with tetratricopeptide repeats 3 [Source:HGNC Symbol;Acc:HGNC:5411] | 6.036199401 | 5.642474947 |
| IFI44 | interferon induced protein 44 [Source:HGNC Symbol;Acc:HGNC:16938] | 6.129397367 | 6.049411827 |
| CD70 | CD70 molecule [Source:HGNC Symbol;Acc:HGNC:11937] | 6.144666241 | 6.266784101 |
| PSMB9 | proteasome subunit beta 9 [Source:HGNC Symbol;Acc:HGNC:9546] | 6.157245233 | 4.897582114 |
| VNN3 | vanin 3 [Source:HGNC Symbol;Acc:HGNC:16431] | 6.17223116 | 6.047809088 |
| BATF2 | basic leucine zipper ATF-like transcription factor 2 [Source:HGNC Symbol;Acc:HGNC:25163] | 6.205528105 | 4.398594717 |
| DDX60 | DExD/H-box helicase 60 [Source:HGNC Symbol;Acc:HGNC:25942] | 6.209767699 | 6.641465194 |
| HERC5 | HECT and RLD domain containing E3 ubiquitin protein ligase 5 [Source:HGNC Symbol;Acc:HGNC:24368] | 6.209916705 | 6.503387261 |
| USP18 | ubiquitin specific peptidase 18 [Source:HGNC Symbol;Acc:HGNC:12616] | 6.244164636 | 5.399186593 |
| HMP19 |  | 6.28666343 | 4.860080618 |
| ATF3 | activating transcription factor 3 [Source:HGNC Symbol;Acc:HGNC:785] | 6.333791881 | 5.679105752 |
| KLHDC7B | kelch domain containing 7B [Source:HGNC Symbol;Acc:HGNC:25145] | 6.395653621 | 6.216389425 |
| RAET1L | retinoic acid early transcript 1L [Source:HGNC Symbol;Acc:HGNC:16798] | 6.481180829 | 4.768153274 |
| ODF3B | outer dense fiber of sperm tails 3B [Source:HGNC Symbol;Acc:HGNC:34388] | 6.498007142 | 7.094414447 |
| SAMD9L | sterile alpha motif domain containing 9 like [Source:HGNC Symbol;Acc:HGNC:1349] | 6.502798636 | 4.861785333 |
| CCL5 | C-C motif chemokine ligand 5 [Source:HGNC Symbol;Acc:HGNC:10632] | 6.525499204 | 6.467314462 |
| MX2 | MX dynamin like GTPase 2 [Source:HGNC Symbol;Acc:HGNC:7533] | 6.558672777 | 4.793692927 |
| HOXB9 | homeobox B9 [Source:HGNC Symbol;Acc:HGNC:5120] | 6.58697131 | 5.442545702 |
| IFIT2 | interferon induced protein with tetratricopeptide repeats 2 [Source:HGNC Symbol;Acc:HGNC:5409] | 6.608860991 | 5.656161848 |
| IFIH1 | interferon induced with helicase C domain 1 [Source:HGNC Symbol;Acc:HGNC:18873] | 6.643791705 | 6.067671433 |
| RTP4 | receptor transporter protein 4 [Source:HGNC Symbol;Acc:HGNC:23992] | 6.718818431 | 5.619486724 |
| CTSS | cathepsin S [Source:HGNC Symbol;Acc:HGNC:2545] | 6.719422363 | 6.249505536 |
| LGALS9 | galectin 9 [Source:HGNC Symbol;Acc:HGNC:6570] | 6.749154594 | 3.432317467 |
| TYMP | thymidine phosphorylase [Source:HGNC Symbol;Acc:HGNC:3148] | 6.757612568 | 6.175912851 |
| CXCL1 | C-X-C motif chemokine ligand 1 [Source:HGNC Symbol;Acc:HGNC:4602] | 6.811940164 | 6.537879442 |
| ISG15 | ISG15 ubiquitin-like modifier [Source:HGNC Symbol;Acc:HGNC:4053] | 6.868190083 | 5.69851221 |
| APOL3 | apolipoprotein L3 [Source:HGNC Symbol;Acc:HGNC:14868] | 6.871192776 | 5.154133811 |
| ETV7 | ETS variant 7 [Source:HGNC Symbol;Acc:HGNC:18160] | 6.880574824 | 5.856103855 |
| APOL1 | apolipoprotein L1 [Source:HGNC Symbol;Acc:HGNC:618] | 6.901286767 | 6.025384246 |
| GBP4 | guanylate binding protein 4 [Source:HGNC Symbol;Acc:HGNC:20480] | 6.918416638 | 6.111731518 |
| CH25H | cholesterol 25-hydroxylase [Source:HGNC Symbol;Acc:HGNC:1907] | 7.061028865 | 6.794489147 |
| IFNB1 | interferon beta 1 [Source:HGNC Symbol;Acc:HGNC:5434] | 7.073044404 | 6.802841198 |
| NEURL3 | neuralized E3 ubiquitin protein ligase 3 [Source:HGNC Symbol;Acc:HGNC:25162] | 7.109328971 | 6.785299113 |
| IFI6 | interferon alpha inducible protein 6 [Source:HGNC Symbol;Acc:HGNC:4054] | 7.286143817 | 7.601159553 |
| SAA2 | serum amyloid A2 [Source:HGNC Symbol;Acc:HGNC:10514] | 7.414181954 | 8.236705347 |
| IFIT1 | interferon induced protein with tetratricopeptide repeats 1 [Source:HGNC Symbol;Acc:HGNC:5407] | 7.439934715 | 6.543715554 |
| HLA-F | major histocompatibility complex, class I, F [Source:HGNC Symbol;Acc:HGNC:4963] | 7.492331599 | 7.775250378 |
| TNFSF10 | tumor necrosis factor superfamily member 10 [Source:HGNC Symbol;Acc:HGNC:11925] | 7.610522404 | 5.558576879 |
| EPSTI1 | epithelial stromal interaction 1 [Source:HGNC Symbol;Acc:HGNC:16465] | 7.733348257 | 6.626434745 |
| SLC15A3 | solute carrier family 15 member 3 [Source:HGNC Symbol;Acc:HGNC:18068] | 7.749251931 | 6.394639446 |
| PDZK1IP1 | PDZK1 interacting protein 1 [Source:HGNC Symbol;Acc:HGNC:16887] | 7.893803624 | 7.370878437 |
| IFNL2 | interferon lambda 2 [Source:HGNC Symbol;Acc:HGNC:18364] | 7.926186319 | 5.437726328 |
| OAS3 | 2'-5'-oligoadenylate synthetase 3 [Source:HGNC Symbol;Acc:HGNC:8088] | 8.50522151 | 7.716879369 |
| CMPK2 | cytidine/uridine monophosphate kinase 2 [Source:HGNC Symbol;Acc:HGNC:27015] | 8.691237172 | 8.056969228 |
| IFITM1 | interferon induced transmembrane protein 1 [Source:HGNC Symbol;Acc:HGNC:5412] | 8.729888012 | 8.73074694 |
| BST2 | bone marrow stromal cell antigen 2 [Source:HGNC Symbol;Acc:HGNC:1119] | 8.765590736 | 9.032292731 |
| MX1 | MX dynamin like GTPase 1 [Source:HGNC Symbol;Acc:HGNC:7532] | 8.821997003 | 7.949106468 |
| OASL | 2'-5'-oligoadenylate synthetase like [Source:HGNC Symbol;Acc:HGNC:8090] | 9.468057318 | 8.77451867 |
| IFI27 | interferon alpha inducible protein 27 [Source:HGNC Symbol;Acc:HGNC:5397] | 9.713450962 | 9.186866255 |
| OAS1 | 2'-5'-oligoadenylate synthetase 1 [Source:HGNC Symbol;Acc:HGNC:8086] | 9.888304699 | 9.142284685 |
| RSAD2 | radical S-adenosyl methionine domain containing 2 [Source:HGNC Symbol;Acc:HGNC:30908] | 9.916252849 | 9.143255761 |
| CXCL11 | C-X-C motif chemokine ligand 11 [Source:HGNC Symbol;Acc:HGNC:10638] | 10.2595425 | 7.603869043 |
| OAS2 | 2'-5'-oligoadenylate synthetase 2 [Source:HGNC Symbol;Acc:HGNC:8087] | 10.74719855 | 10.02353622 |
| XAF1 | XIAP associated factor 1 [Source:HGNC Symbol;Acc:HGNC:30932] | 10.7472847 | 9.605353782 |
| CXCL10 | C-X-C motif chemokine ligand 10 [Source:HGNC Symbol;Acc:HGNC:10637] | 10.75521378 | 8.581547815 |
